# Maintaining diversity in structured populations

**DOI:** 10.1093/pnasnexus/pgaf252

**Published:** 2025-08-08

**Authors:** David A Brewster, Jakub Svoboda, Dylan Roscow, Krishnendu Chatterjee, Josef Tkadlec, Martin A Nowak

**Affiliations:** John A. Paulson School of Engineering and Applied Sciences, Harvard University, Boston, MA 02134, USA; Department of Molecular and Cellular Biology, Harvard University, Cambridge, MA 02138, USA; Institute of Science and Technology Austria, Klosterneuburg 3400, Austria; Department of Mathematics, University of Illinois at Urbana-Champaign, Urbana, IL 61801, USA; Institute of Science and Technology Austria, Klosterneuburg 3400, Austria; Computer Science Institute, Charles University, Prague 116 36, Czech Republic; Department of Organismic and Evolutionary Biology, Harvard University, Cambridge, MA 02138, USA; Department of Mathematics, Harvard University, Cambridge, MA 02138, USA

**Keywords:** evolutionary dynamics, diversity, graphs, random walk, moran process

## Abstract

We examine population structures for their ability to maintain diversity in neutral evolution. We use the general framework of evolutionary graph theory and consider birth–death (bd) and death–birth (db) updating. The population is of size *N*. Initially all individuals represent different types. The basic question is: what is the time $T_{N}$ until one type takes over the population? This time is known as consensus time in computer science and as total coalescent time in evolutionary biology. For the complete graph, it is known that $T_{N}$ is quadratic in *N* for db and bd. For the cycle, we prove that $T_{N}$ is cubic in *N* for db and bd. For the star, we prove that $T_{N}$ is cubic for bd and quasilinear (NlogN) for db. For the double star, we show that $T_{N}$ is quartic for bd. We derive upper and lower bounds for all undirected graphs for bd and db. We also show the Pareto front of graphs (of size N=8) that maintain diversity the longest for bd and db. Further, we show that some graphs that quickly homogenize can maintain high levels of diversity longer than graphs that slowly homogenize. For directed graphs, we give simple contracting star-like structures that have superexponential time scales for maintaining diversity.

Significance Statement Evolution—either by genetic reproduction or by learning—occurs in populations. The structure of a population affects the time scale and outcome of evolutionary processes. The propensity of populations to maintain diversity is of great interest in evolutionary biology, ecology, and social science. Here, we calculate for how long various population structures can maintain diversity under neutral evolution. In this setting, diversity is lost by random drift. We give precise results for a large variety of structures. We find that some structures have higher-order polynomial or even superexponential timescales for maintaining diversity. For realistic population sizes of thousands or millions of individuals, those structures can maintain diversity for times that exceed the lifetime of a universe. Therefore, they protect diversity “forever.”

## Introduction

Evolutionary graph theory is a method for studying the effect of population structure on evolutionary dynamics (1–9). The individuals occupy the vertices of graphs, and the edges specify interactions between individuals. The special case of a well-mixed population is given by a complete graph with identical weights. In the case of constant selection, we are interested in the role of population structure on suppression/amplification selection effects and evolutionary timescales (10–21). Weighted edges can create amplification or suppression effects (15, 22, 23). Isothermal graphs have the same fixation probability as the well-mixed population (3, 4, 24). Unidirectional edges can introduce exceedingly long absorption times (25, 26). Even in neutral evolution, details of the evolutionary process can heavily affect timescales (27–29). Environments with mixed resource abundances have effects on fixation probabilities (30, 31). For frequency dependent selection, it is known that some graphs and update rules can promote evolution of cooperation (6, 32–44). Expected absorption times in structured populations have also been studied in continuous time (45).

In this article, we analyze graphs for their ability to maintain diversity in neutral evolution. We consider a population of finite size, *N*. Initially all individuals represent different types. All types have the same reproductive rate. We ask: what is the expected time, $T_{N}$, until all individuals descend from the same type. This time is known as total coalescence time in biology. For a well-mixed population with *N* individuals, the coalescence time is known to be *N* generations, or $N^{2}$ reproductive events (46–48). Our work builds upon and extends prior studies of absorption times in evolutionary dynamics. Iwamasa and Masuda (27) study the consensus time of voter models on various graphs. They show that for two types on small networks, the barbell and double star graph families maximize the expected absorption time for death–birth (db) and birth–death (bd) updating, respectively. Further, they calculate the asymptotic expected absorption times for two types on those graphs. Diaz et al. (25) present a directed graph family that has at least exponential absorption time if one of the two types has a fitness advantage. Gao et al. (29) analyze all undirected graphs of size N=6. They compute the absorption time starting with two types under neutral evolution and db updating. They find that graphs with a bottleneck between two large components typically have a large absorption time.

In contrast, our study expands the scope to *N* types and provides rigorous analyses of the fastest and slowest graphs. We also examine directed graphs, which can superexponentially broaden the diversity timescales. We give upper and lower bounds for the diversity time of any undirected or directed graph. In addition to our proofs for arbitrarily sized population structures, we analyze various evolutionary properties of graphs up to size N=100. In particular, we are concerned not only with the expected time until homogeneity but also the expected number of types remaining in the population as a function of time. We examine tradeoffs in diversity time between db and bd updating relative to the population structure.

## Results

### Diversity in structured populations

Consider a population of *N* individuals. Initially each individual is of a different type. Every time step, one individual is selected for birth and one individual for death. The individual selected for death is removed from the population. The individual selected for birth creates a copy of itself at the location of the individual that was selected for death. After many steps, the population will become homogeneous, which means that all individuals are of the same type. Once the population is homogeneous it remains so. Thus, homogeneity is an absorbing state. In principle, there are *N* different absorbing states—one for each of the types that are present initially. We are interested in calculating the average time $T_{N}$ until one of the absorbing states is reached.

Population states with more than one type are called heterogeneous (or diverse). All heterogeneous states are transient. They will be lost after some time. The absorption time $T_{N}$ gives us a measure for the ability of a population structure to maintain diversity.

We are interested in exploring population structures for their ability to maintain diversity for extended periods of time. We describe the population structure as a strongly connected directed graph G=(V,E). The vertices *V* denote the locations of individuals in the population. The edges *E* represent possible interactions between the individuals. For any two individuals, *u* and *v*, if *v* is a neighbor of *u* in *G*, then the offspring of *u* can replace *v*. The evolutionary dynamics on graphs can be interpreted as biological reproduction or learning. In the case of learning, one individual becomes a learner and the other a teacher. Then, the learner adopts the type of the teacher.

The order of birth and death matters. Under birth–death (bd) updating, an individual *i* is chosen uniformly at random from the population to reproduce. Then, an individual *j* is chosen for death uniformly at random from the outgoing neighbors of *i*. If individual *i* resides at location u∈V and *j* resides at location v∈V, then the probability of this event occurring at any step given (u,v)∈E is


(1) $$\frac{1}{N}\cdot \frac{1}{\deg^{+}\;(u)}.$$

Here, $\deg^{+}\;(u)$ represents the number of outgoing neighbors of vertex *u*.

Under death–birth (db) updating, an individual *j* is chosen uniformly at random from the population to die. Then, an individual *i* is chosen for birth uniformly at random from the incoming neighbors of *j*. If individual *j* resides at location v∈V and *i* resides at location u∈V, then the probability of this event occurring at any step given (u,v)∈E is


(2) $$\frac{1}{N}\cdot \frac{1}{\deg^{-}\;(v)}.$$

Here, $\deg^{-}\;(v)$ represents the number of incoming neighbors of *v*.

We call a graph undirected (bidirectional) if (u,v)∈E implies (v,u)∈E for all vertices u,v∈V. In other words, individuals have reciprocal interactions in undirected graphs. Since the number of incoming neighbors is the same as the number of outgoing neighbors in an undirected graph, we denote the number of neighbors of a vertex u∈V in an undirected graph as simply deg(u).

An undirected graph where all vertices have the same number of neighbors is called a regular graph. Suppose all vertices in a regular graph have *D* neighbors. Then assuming (u,v)∈E, the probability that location u∈V is selected for birth and location v∈V is selected for death is given by


(3) $$\frac{1}{N}\cdot \frac{1}{D}.$$

**Fig. 1.**
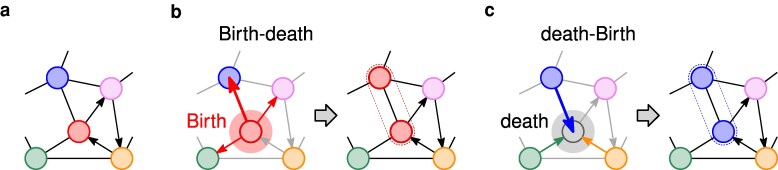
a) An example of a directed graph with various types in the population. b) For bd updating, first an individual is chosen for reproduction and then one of its neighbors is chosen to be replaced; here the center vertex is selected for birth and the topmost vertex is selected for death. c) For db updating, first an individual i chosen for death (or to update its type) and then one of the neighbors is chosen for reproduction; here the center vertex is selected for death and the topmost vertex is selected for birth.

Since this relationship holds regardless of the update rule, questions about diversity on regular graphs are unaffected by the governing dynamics. See Fig. 1 for illustrations of the two update rules.

### Time of evolution

**Fig. 2.**
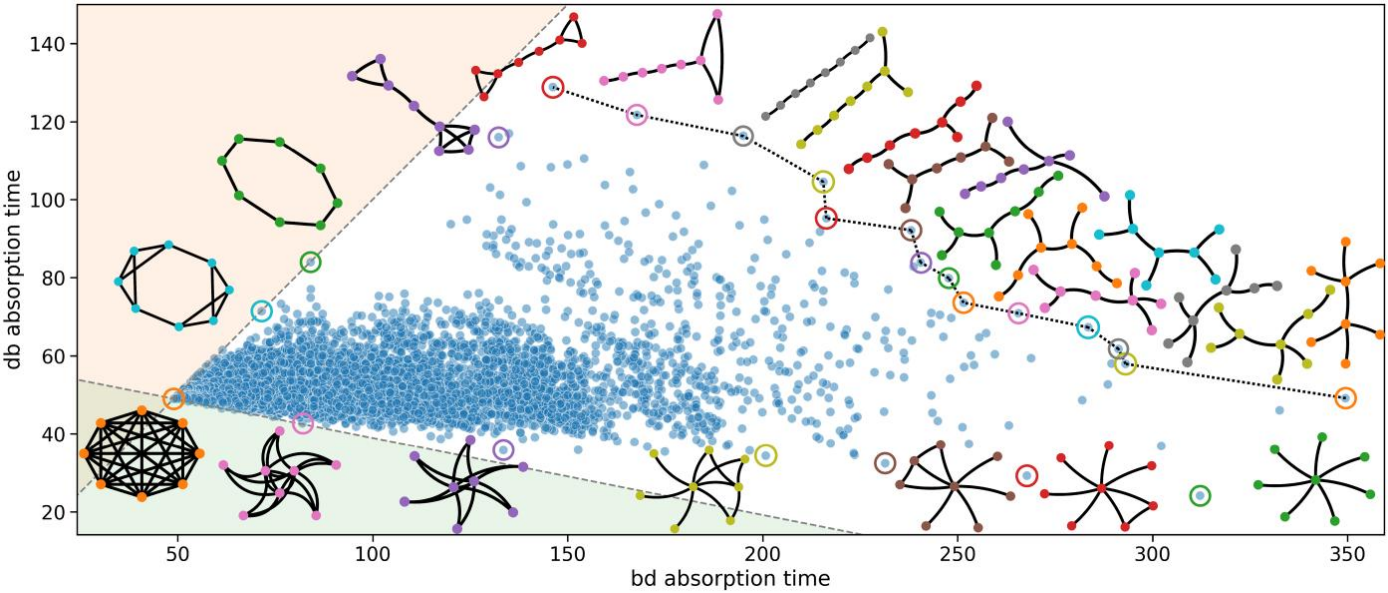
Absorption times for bd vs db updating for all 11,117 connected undirected graphs with N=8 vertices. Each dot represents a graph. The positively sloped dashed line has unit slope and passes through the dot representing the complete graph; all regular graphs are on this dashed line. The negatively sloped dashed line is such that it is the smallest sector that contains all the dots and has apex at the dot that corresponds to the complete graph. Some dots are circled with its corresponding graphical representation displayed adjacently. The double star maximizes the bd absorption time. The barbell maximizes the db absorption time. The Pareto front (the dashed line segments) connects the two.

**Fig. 3.**
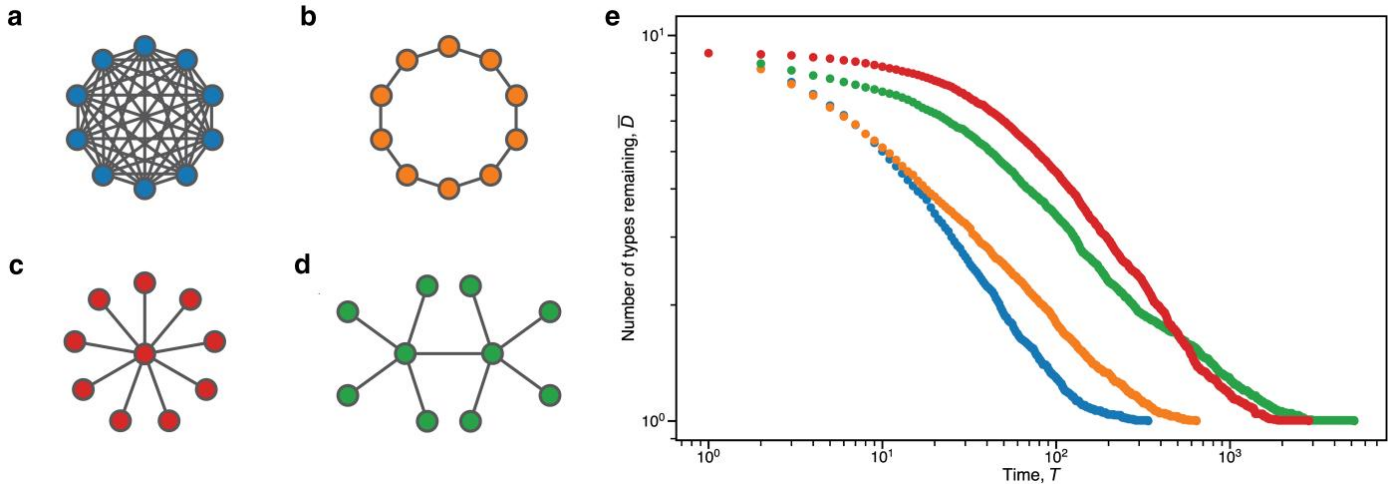
Various undirected graph families on N=10 vertices: a) the complete graph; b) cycle; c) star; and d) double star. e) Results of average number of types remaining in the population, $\bar{D}$, at time *T*, averaged over 250 simulations of bd updating per graph. Each graph has N=10 vertices. The plot is on a log–log scale to accentuate the number of types remaining when the values are close to one another. The horizontal axis begins at T=1. If $\bar{D}=1$ at a particular time *T*, no dot is drawn. Note that on average the star graph maintains more diversity than the double star graph up until roughly $T\approx 5\times 10^{2}$. See Fig. S1 in the SI Section 11 for the plot when N=100.

We want to calculate the time (in number of steps) until the population becomes homogeneous. Since the population starts with maximum diversity, we measure the ability of a population structure to maintain diversity by the expected time until homogeneity is reached. We refer to the time until homogeneity as the absorption time. It is important to note that the absorption time of an observed bd or db process is a number while the absorption time of a population structure is a random variable. We are interested in the expected absorption time of various population structures (see Figs. 2 and 3 and Table 1 for a few examples).

**Table 1.** Asymptotic absorption times for various graph families under bd and db updating.

	Graph family	bd time	db time
Undirected	Complete	$\Theta (N^{2})$	$\Theta (N^{2})$
	Cycle	$\Theta (N^{3})$	$\Theta (N^{3})$
	Star	$\Theta (N^{3})$	Θ(NlogN)
	Double star	$\Theta (N^{4})$	–
	Barbell	–	$\Omega (N^{4})$
	Undirected graph	$O(N^{6}\log\;N)$ , Ω(NlogN)	$O(N^{5}\log\;N)$ , Ω(NlogN)
Directed	Contracting star	$2^{\Theta (N\log\;N)}$	$2^{\Theta (N\log\;N)}$
	Directed graph	$2^{O(N\log\;N)}$	$2^{O(N\log\;N)}$ , Ω(NlogN)

We use *O*, *Ω*, and *Θ* to represent asymptotic upper, lower, and tight bounds, respectively (see Section 1.2 of (49) for formal definitions). For the double star, we have no estimate for db updating. For the barbell, we have no estimate for bd updating. For bd updating, there is a gap between the slowest undirected family of graphs we know (double star) and our theoretical upper bound for any undirected graph. Similarly, for db updating there is a gap between the slowest undirected family of graphs we know (barbell) and our theoretical upper bound for any undirected graph. In both cases, whether our analysis is not tight enough or there are even slower graph families that we have not found is unknown. In contrast, we find that contracting stars are the slowest of the directed graphs.

We measure time as a function of the population size *N*. Often, we are interested in the asymptotics of the time rather than an exact expression.

### Well-mixed populations

Consider a population structure with *N* individuals where every individual interacts with every other individual. This configuration is assumed when analyzing evolutionary dynamics without explicit reference to population structure. We represent this well-mixed population structure as a complete graph on *N* vertices with self-loops: the edges are the Cartesian product of the vertex set by itself. Complete graphs are highly symmetric. Thus, knowledge about the frequencies of the various types in the populations is sufficient information for calculating the expected absorption time.

Consider bd updating and suppose there are currently *k* types in the population. Let $\lambda \equiv (\lambda_{1},\ldots ,\lambda_{k})$ denote the vector of abundances. We have $\lambda_{1}+\cdots +\lambda_{k}=N$. We order the abundances such that $\lambda_{1}\geq \lambda_{2}\geq \cdots \geq \lambda_{k}>0$.

During each time step, one of three events occurs:

(1) the abundances, $\lambda_{1},\ldots ,\lambda_{k}$, remain exactly the same;
(2) the abundance of one type increases by one, while the abundance of another type decreases by one, but the number of types in the population remains the same;
(3) the abundance of one type increases by one, the abundance of another type decreases by one, and the number of types in the population decreases by one.

The process can be described as beginning in a state of maximum entropy ($\lambda_{1}=\cdots =\lambda_{N}$) and reaching a state of minimum entropy ($\lambda_{1}=N$). We show that the expected absorption time of this process starting from configuration *λ* is exactly


(4) $$T_{N}=N^{2}-N-\sum_{i=1}^{k}\sum_{\ell =1}^{\lambda_{i}-1}\frac{(N+\lambda_{i}-2\ell )\ell }{N-\ell }.$$

One intuitive way to think about the formula is as follows (see SI Section 2). Imagine the “histogram” of the partition *λ* and for each h≥0, denote by $b_{h}$ the number of boxes above the line y=h. (In particular, $b_{0}=N$.) An explicit formula for $b_{h}$ is $\sum_{i}\max(\lambda_{i}-h,0)$. Then, the expected absorption time from the given configuration is


(5) $$T_{N}=N\cdot \left(N-\sum_{h=0}^{N-1}\frac{b_{h}}{N-h}\right).$$

In the case when k=N (i.e. $\lambda_{1}=\cdots =\lambda_{N}$), Eq. 4 yields the expected absorption time from maximum diversity as


(6) N⋅(N−1).

If we consider a complete graph with no self-loops, the expected absorption time is $(N-1{)}^{2}$ which is not asymptotically different than the expected absorption time with self-loops. (See the SI Section 2 for details.) Well-mixed population structures are represented by regular graphs since each vertex has the same number of neighbors. Thus, our results under bd dynamics are the same as the results for db updating. Next, we will explore population structures beyond well-mixed populations.

### Cycles

Consider a population with *N* individuals whose locations form a circle-like structure. Individuals interact with each of their two adjacent neighbors. This population structure is represented by an undirected cycle graph. A cycle is a regular graph since each vertex has exactly two neighbors. Types are always clustered together on the vertices of the cycle. As individuals give birth and die, some clusters take over others. In many steps of the selection process on the cycle, individuals in the interior of the cluster are chosen for reproduction; the individual is only able to reproduce to locations where individuals of its type already reside. Thus, no change in the population configuration occurs. When the individual selected for birth resides on a boundary between differing types, there is a 50% chance that the configuration of the population changes. These updates are called active steps.

Individuals of the same type are always clustered together on the cycle. Using similar logic to the case of the well-mixed population, it suffices to know only the frequencies of the types and their relative locations around the perimeter of the cycle. Recall $b_{h}=\sum_{i}\max(\lambda_{i}-h,0)$. For the expected absorption time from given frequencies, we arrive at


(7) $$T_{N}=\frac{\left(N+1)N(N-1\right)}{6}-\sum_{h=0}^{N-1}b_{h}\cdot h.$$

When the process starts with maximum diversity, this results in the expected absorption time as simply


(8) $$T_{N}=\frac{\left(N+1)N(N-1\right)}{6}.$$

Some existing results for cycles are known (50). See the SI Section 3 for more details.

So far, we have examined regular graphs. Next, we will analyze graphs that are far from regular.

### Stars

A star graph has one central vertex and multiple vertices connected to the central vertex. For a star with *N* vertices we denote *n* as the number of noncentral vertices so that n+1=N. More formally, the central vertex c∈V is connected to the remaining vertices $v_{1},\ldots ,v_{n}\in V$ bidirectionally. These graphs are not regular because the central vertex has degree *n* whereas the remaining vertices have degree 1. However, stars are very similar to complete graphs in the following sense: the noncentral vertices are connected to each other with paths of length two. There are two types of events that can occur on a star:

1. a vertex on the periphery is selected for birth, or
2. the central vertex is selected for birth.

First, consider bd updating on a star. The case of event 1 occurs with probability 1−1/N. The only place for a vertex on the periphery to give birth is into the center. On the other hand, the case of event 2 happens with probability only 1/N. When the center is selected for reproduction, it places its offspring at a vertex chosen uniformly at random from the periphery. On average, the center reproduces every *N* steps. The type of the individual at the central vertex at the time it gives birth is highly likely to be directly proportional to the relative abundances of the types on the periphery. Thus the process is akin to the complete graph with each step scaled by a factor of *N*. We show that the expected absorption time of a star on *N* vertices is $\Theta (N^{3})$. We conjecture that the exact expected absorption time under bd updating is


(9) $$T_{N}=n^{3}-n^{2}+n\cdot H_{n}$$

The expression $H_{n}$ represents the sum of the first *n* terms of the harmonic series $\frac{1}{1}+\frac{1}{2}+\frac{1}{3}+\cdots$. See the SI Section 5 for more details.

Next, consider db updating on a star. The case of event 1 only happens when the central vertex is selected for death. This event occurs with probability 1/N. On the other hand, event 2 occurs with remaining probability. Let *i* be the number of types in the periphery different from the center. There are two kinds of active steps. Either the center is replaced by a different type or the center reproduces onto a different type. The former event has probability (1/N)⋅(i/n) and the latter event has probability i/N. The ratio between the two events is 1/n. Thus in *n* active steps, the center is not replaced by a different type with probability


(10) $${\left(1-\frac{1}{N}\right)}^{n}\geq 1/e.$$

If the center is replaced, we restart the process. That means with constant probability, the process ends in *n* active steps. Counting all steps gives a logarithmic slowdown yielding an expected absorption time of Θ(NlogN) under db dynamics. See the SI Section 5 for details.

Stars promote diversity under bd updating and demote diversity under db updating.

### Double stars

A double star graph is a bidirectional graph composed by joining two equally sized stars together by their central vertices. For simplicity, we consider only double stars with an even number of vertices. We denote *n* as the number of noncentral nodes on one star. The total number of nodes in a double star is N=2n+2. Similar to stars, double stars are also nonregular graphs since the two central vertices have degree n+1=N/2 and the remaining nodes have degree 1. We consider bd updating. Compared to the star, there is an additional event that could happen: one center could give birth onto the other center. This invasion attempt happens with probability roughly


(11) $$2\cdot \frac{1}{N}\cdot \frac{1}{N/2}\;=\Theta \left(N^{-2}\right).$$

Suppose a star has a homogeneous population except for a single differing type at its central vertex. It is known that under bd updating, the probability the individual initially placed at the center will take over the population is


(12) $$\frac{\frac{1}{n}}{\frac{1}{n}+n}=\;\frac{1}{1+n^{2}}\;=\Theta \left(N^{-2}\right).$$

This probability is known as the fixation probability (see Ref. (4)). Successful invasion is rare. If the individual initially placed at the center goes extinct, this likely happens quickly, in a constant number of steps. Since both an invasion attempt and fixation must occur, a successful invasion takes roughly $\Theta (N^{4})$ steps on average. Thus, the typical evolution of the population on a double star proceeds as follows:

1. Evolution occurs primarily in the two stars of the double star; sometimes invasion attempts occur but invaders are quickly wiped out.
2. After roughly $\Theta (N^{3})$ steps the stars are each homogeneous, but there are still two types remaining in the population.
3. The process terminates after the next successful invasion; this takes on average $\Theta (N^{4})$ steps.

Overall, the expected absorption time for a double star is $\Theta (N^{4})$. This structure promotes diversity under bd updating for the longest out of all of the undirected graphs we considered. In db updating, the central hubs give birth often. Though unlike the star, the two stars on the double star must eventually agree on the type. See the SI Section 7 for proofs of the upper and lower bounds.

### Barbells

**Fig. 4.**
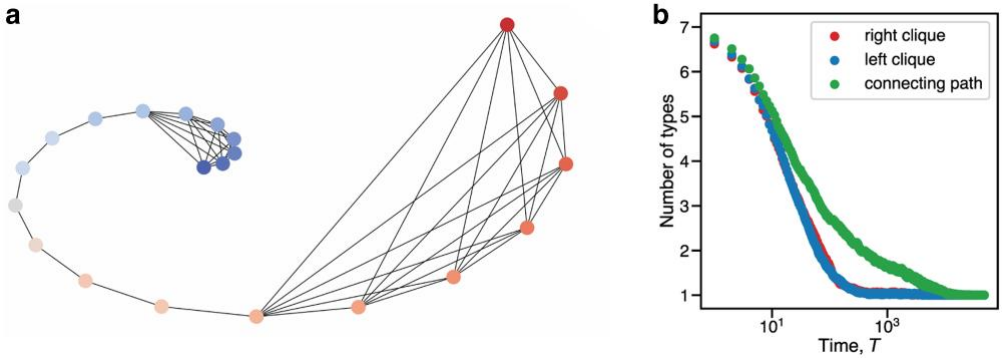
a) A barbell graph with N=7+7+7=21 vertices. b) A semilog plot displaying the number of types in each part of the barbell graph vs. time, averaged over 250 simulations.

A barbell graph is a bidirectional graph composed by joining two equally sized cliques (i.e. fully connected subgraphs) together by a path. If the two cliques have *n* vertices, then the path has *n* vertices, combining for a total of N=3n vertices in the graph (see Fig. 4).

For db updating, the process eventually settles on two types in the population. Each type occupies a clique and part of the path. Then one type attempts to invade the other clique. There is a 1/n chance invasion is successful. But invasions only happen roughly every $n^{3}$ steps due to the absorption time of a path graph. The ends of the paths have high degree. Thus it is much more likely that the path remains heterogeneous when it is nearly homogeneous due to the db updating process. The process resolves in expected time $\Omega (N^{4})$. See the SI Section 8 for details. For bd updating, the barbell diversity time is faster. Invasion into a clique happens at a much higher rate for bd updating since an end node on the connecting path has a 1/2 probability of invading if it selected for birth (with probability 1/N). However for db updating, invasion occurs when a node in a clique connected to the connecting path dies (with probability 1/N); but, there is only a 1/n probability that the node on the path will invade into the clique.

### Time bounds on any two-way population structure

Under bd updating, it is known that if the initial configuration on an undirected graph contains only two types, the expected absorption time is $O(N^{6})$. Recent work for a multitype bd process yields an $O(N^{7})$ upper bound for the expected absorption time when the process starts with *N* types (51). We show that this upper bound can be tightened to $O(N^{6}\log\;N)$ by a divide-and-conquer proof strategy. For db updating, the literature on consensus problems gives an $O(N^{5})$ upper bound on the expected absorption time when the process starts with two types (52). Similar to the bd case, we can achieve an upper bound on the expected absorption time for any graph of $O(N^{5}\log\;N)$ for db updating. See the SI Section 9 for details.

For both bd and db updating, an Ω(NlogN) lower bound for the expected absorption time follows by considering that at least N−1 locations must eventually be a death site for the process to absorb. The expected amount of time for N−1 locations to be a death site is $(N-1)\cdot H_{N-1}$. We note that $H_{N-1}=\Theta (\log\;N)$. See Methods and SI Section 9 for more details and proof.

### Contracting star

We have shown that all undirected graphs have expected absorption time at most some polynomial function of the population size. We call these absorption times short. In neutral evolution under bd updating, it is known that some families of directed graphs (graphs that have some one-way connections) also have short absorption times (26). We give a construction of a directed graph family with long absorption times, times that are some superexponential function of the population size.

**Fig. 5.**
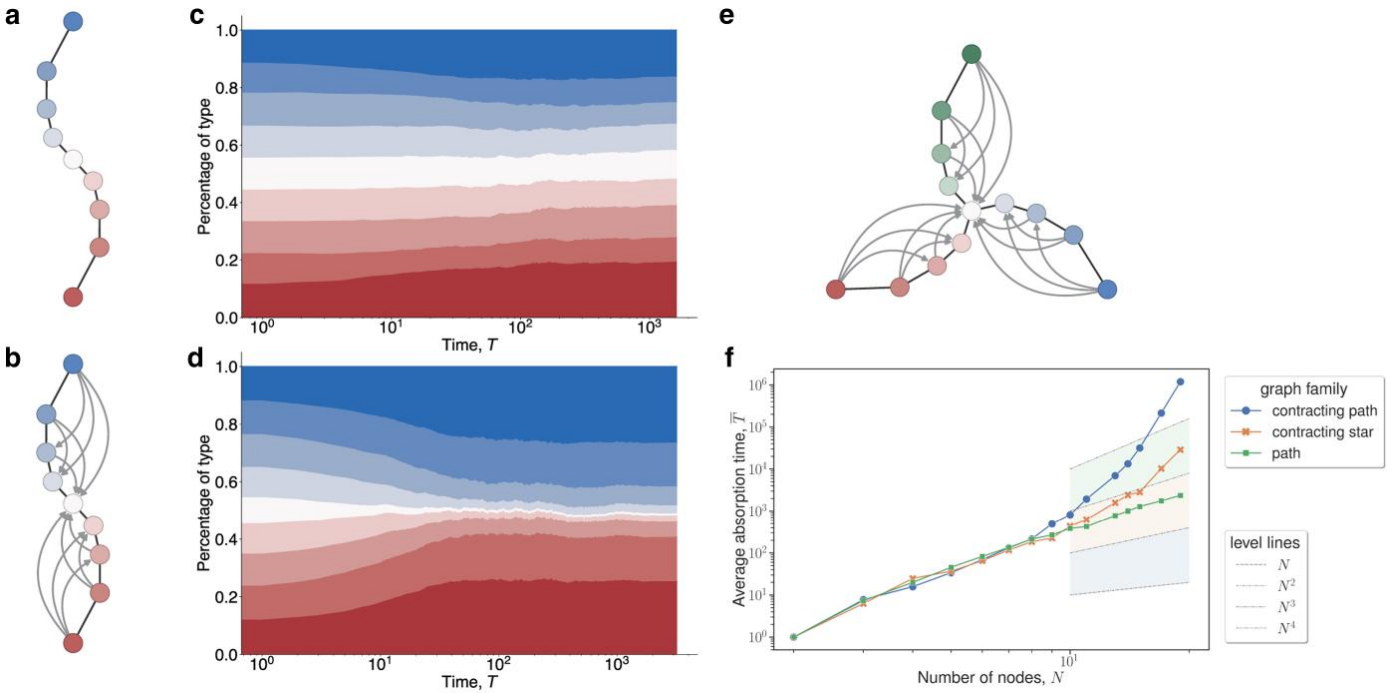
a) An undirected path on N=9 vertices. b) A contracting star (or contracting path) on N=9 vertices and b=2 blades. A contracting path is a composition of a bidirectional (undirected) path and “contracting” directed edges pointing inwards. c) Plots for the normalized frequency of a type in the population of the undirected path on N=9 vertices vs. time over 1,000 simulations. The population starts with the types as colored in (a). The height of each color is proportional to frequency of the corresponding type in the population. d) Plots as in c) but for the contracting path on N=9 vertices. e) Contracting star on N=13 vertices and b=3 blades. f) Simulation results of the expected absorption time for bd updating of a undirected path, a contracting path, and a contracting star (with three blades) over varying population sizes. The plot is semilog and *N* ranges from 2 to 20. Each dot represents the average of 100 trials starting from maximum diversity. The dotted lines towards the right side of the plot indicate various power law level lines. The path graph family follows a level line, but the contracted star graph families grow faster than some polynomial of the population size.

A contracting star is a directed graph with multiple blades bidirectionally connected to a central vertex. Each blade consists of a bidirectional path. For every pair of vertices on a blade, there is a directed edge from the vertex farther from the center to the vertex closer to the center (see Fig. 5).

For simplicity, we restrict contracting stars to have *N* vertices and *b* equally sized blades so that *b* divides N−1. We show that under bd updating, the expected absorption time of a contracting star with two blades is


(13) $$T_{N}\geq 2^{\Omega (N\log\;N)}.$$

See the SI Section 10 for more details.

### Computer experiments

**Fig. 6.**
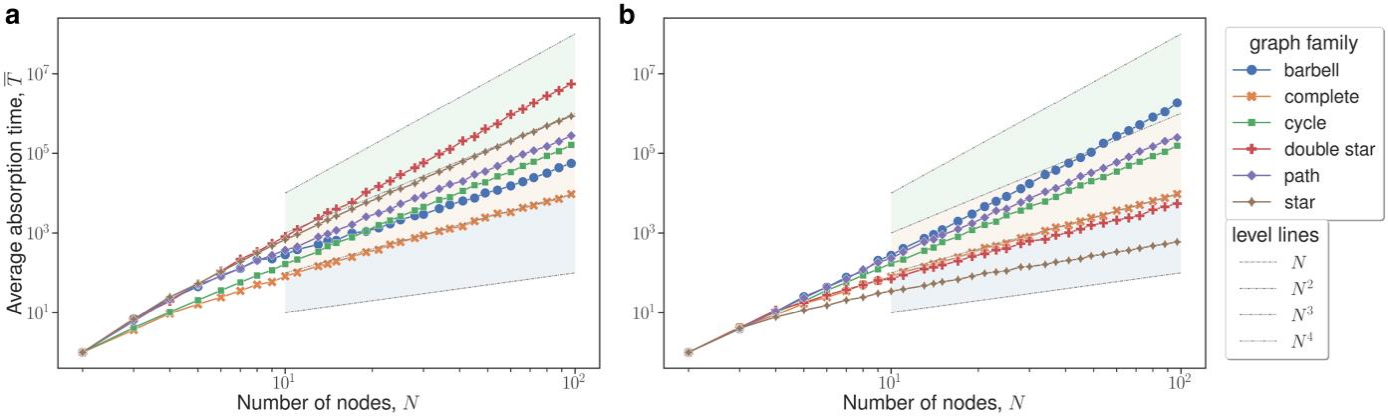
Log–log plots of the absorption time (*T*) of various graph families for 1≤N≤100, with 250 simulations per data point. The boundaries of the shaded regions denote the power law exponent; these level lines are described in the legend on the bottom right. We can see that regular graphs (e.g. complete and cycle graphs) are unaffected by the updating dynamics. a) bd updating b) db updating.

We investigate properties of small graphs. First, we consider various graph families with N≤100 vertices and estimate the expected absorption times via simulations. For bd updating (see Fig. 6a), we see that double stars are asymptotically slower than stars, which is in alignment with our theoretical results.

Similarly, stars seems asymptotically slower than cycles and paths. Finally, cycles and paths seem asymptotically slower than well-mixed populations. It appears that paths are slower than cycles by some multiplicative constant. In contrast, for db updating (see Fig. 6b), we see that double stars are not the asymptotically slowest graphs presented. We do not rigorously analyze double stars under db updating, but from Fig. 6b we see that the diversity time of the double star becomes much faster under db updating compared to bd updating. We also see that stars absorb faster than complete graphs. It is known that the absorption times for regular graphs (e.g. complete graph, cycles) are independent of the two updating mechanisms we consider.

**Fig. 7.**
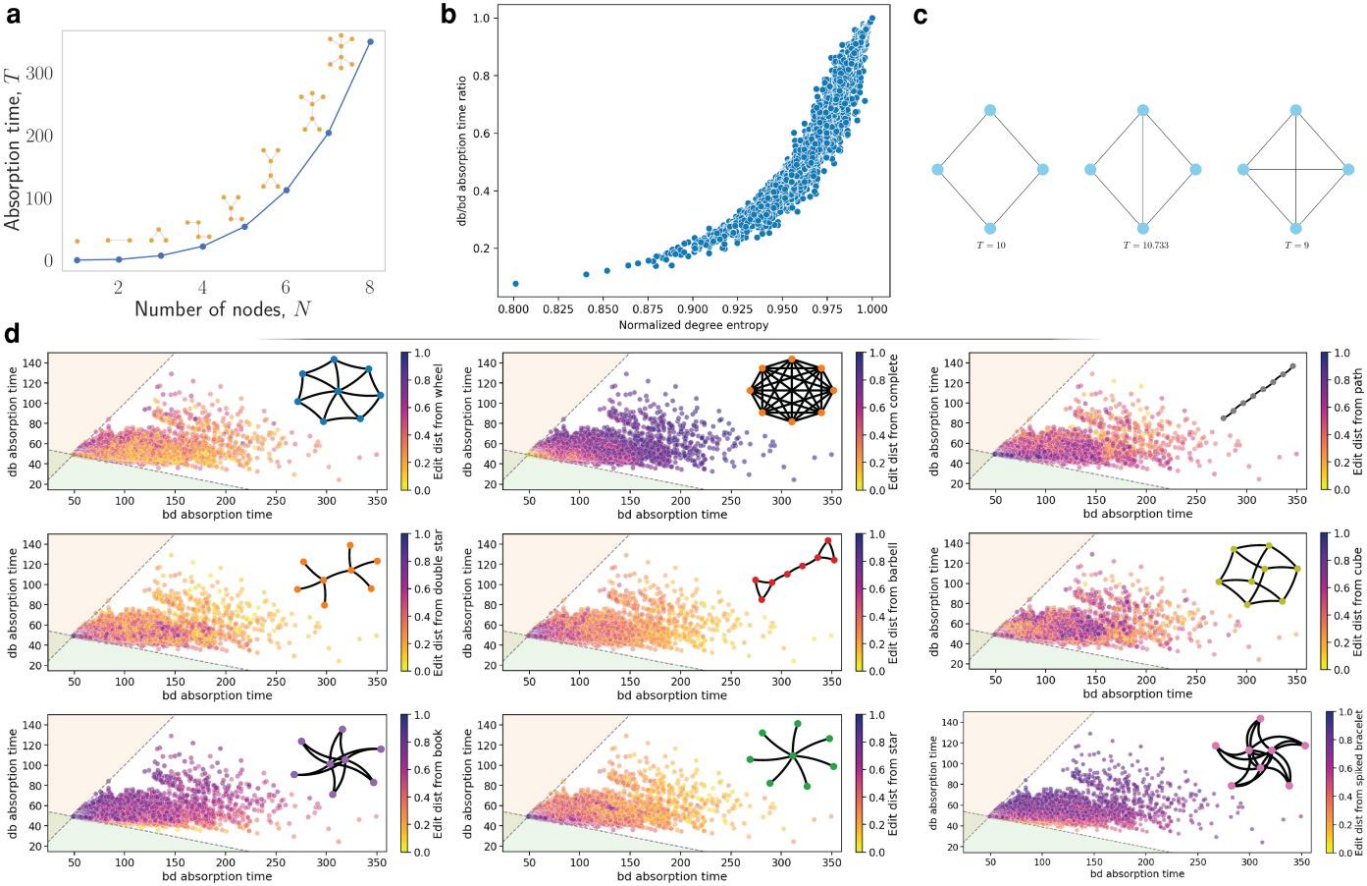
a) Absorption times (*T*) of the slowest undirected graphs of bd updating for 1≤N≤8. b) db absorption time divided by bd absorption time (db/bd absorption time ratio), vs. normalized degree entropy for all graphs with N=8 vertices. c) Three graphs on N=4 vertices with corresponding bd absorption times below. We see that adding an edge can increase the absorption time (compare the leftmost graph absorption time to that of the center graph). We also see that adding an edge can decrease the absorption time (compare the center graph absorption time to that of the rightmost graph). d) Absorption times under bd and db updating for N=8. The colors of the dots indicate the graph edit distance from a target graph. Lighter colors indicate a closer distance to the target whereas darker colors indicate a farther distance.

Next, we look at all connected undirected graphs with N=8 vertices. We analyze the bd absorption time vs. the db absorption time for such graphs. From Fig. 2, we observe that there are no graphs that have a higher db absorption time than bd absorption time; the graphs that come closest are the regular graphs for which the two times are exactly equal. The edit distance between two graphs is the minimum number of vertex (or edge) deletions (or insertions) to transform one graph into an isomorphic version of the other. The Pareto front of the graphs with the longest absorptions under bd or db seems to fall under a gradient from barbell to double star, with small graph edit distance between consecutive members. We also see that distance in the bd vs db absorption time plane is not always correlated with the graph edit distance (see Fig. 7d). The normalized degree entropy of an undirected graph is defined as


(14) $$-\;\frac{1}{\log\;N}\sum_{u\in V}\frac{\deg\;(u)}{\sum_{v\in V}\deg\;(v)}\;\log\;\left(\frac{\deg\;(u)}{\sum_{v\in V}\deg\;(v)}\right)$$

The normalized degree entropy measures the regularity of the graph; the value of this quantity is between 0 and 1. In Fig. 7b, we see that low normalized degree entropy roughly correlates to lower db absorption times vs. bd absorption times. For N=8, the graph with the lowest normalized degree entropy is the star. Graphs with higher normalized degree entropy are more time-robust to the particulars of the two updating mechanisms. We note that adding edges to a graph does not necessarily decrease its expected absorption time (see Fig. 7c).

For all connected undirected graphs with N≤8, we computed the exact expected absorption time under bd updating. We found that the slowest absorbers are graphs resembling double stars (see Fig. 7a). This may indicate that double stars are the slowest absorbers under bd updating.

Finally, we more closely examine bd updating on various graphs of the same size. We are interested in the average number of type remaining at time *T* in the process. We find that although a double star has a longer expected absorption time, a star can maintain more types for a longer amount of time (see Fig. 3).

## Discussion

In summary, we have shown that population structure and update rules can have large effects on the maintenance of diversity.

We have shown the following five results which hold both for bd and for db updating: (i) For the complete graph, which describes a well-mixed population, the time scale for loss of diversity is $\Theta (N^{2})$. (ii) For the cycle, which describes a simple 1D population structure, the time scale is $\Theta (N^{3})$. (iii) The lower provable bound for any undirected graph is Ω(NlogN); for db updating the star matches this lower bound but we have no matching example for bd updating. (iv) The slowest directed graph which we have identified so far—the contracting star—loses diversity at the vast time scale of $2^{\Theta (N\log\;N)}$. (v) The upper bound for the directed graph is $2^{O(N\log\;N)}$.

We derive the following additional results which hold for bd updating: (i) the star has time scale $\Theta (N^{3})$; (ii) the double star has time scale $\Theta (N^{4})$; (iii) for any undirected graph the upper bound is $O(N^{6}\log\;N)$.

We derive the following additional results which hold for db updating: (i) the star has time scale Θ(NlogN); (ii) the barbell has a lower bound of $\Omega (N^{4})$; (iii) for any undirected graph the upper bound is $O(N^{5}\log\;N)$.

For bd updating, we establish that double-star graphs have a time scale of $\Theta (N^{4})$,while any undirected graph has an upper bound of $O(N^{6}\log\;N)$. Closing this gap remains an open challenge. Furthermore, the complete graphs has a time scale of $\Theta (N^{2})$ while the lower bound for any graph is Ω(NlogN). Determining whether tighter bounds can be achieved for intermediate cases is an intriguing direction for future research. While we prove that star graphs have diversity times of $\Theta (N^{3})$, we conjecture an exact formula for their diversity time. Proving this formula would provide a deeper understanding of star dynamics.

For db updating, we show that barbell graphs have a lower bound of $\Omega (N^{4})$ and that any graph has an upper bound of $O(N^{5}\log\;N)$. Closing this gap remains an open challenge. Star graphs have an upper bound of O(NlogN) matching the general lower bound of Ω(NlogN) for any graph.

The superexponential time scale that is achieved by contracting stars means that diversity can by maintained “forever” if the population size is not too small. But even the $N^{4}$ time scale that is reached by undirected graphs, which corresponds to $N^{3}$ generations, would imply that for (microbial) population sizes of $N=10^{6}$ diversity is maintained for $10^{18}$ generations, which exceeds the time scale of evolution on earth.

Our study suggests many possibilities for future research. We have analyzed the expected time until a population becomes homogeneous. However, we noticed that a graph could maintain some number of types longer than another but still homogenize quicker. For example, we observed that a double star becomes homogeneous slower than a star on average, but a double star loses types more rapidly than a star at times closer to the inception of the process. It would be insightful to understand which population structures can maintain diversity of at least a certain number of distinct types for the longest.

Also, the notion of diversity which we have used here is only one possibility of many. We have counted the number of distinct types that is present in the population. Other notions of diversity—such as the Simpson index or the Shannon entropy—take into account the frequency of different types and/or the spatial clustering of types.

We plan to study the effect of mutation on maintaining diversity. In this setting, new types are produced by mutation and existing types become extinct by random drift. Then the population reaches a steady state level of diversity. We ask: how does population structure affect diversity at steady state.

Finally, one should investigate how variation in fitnesses affects our results. Our work in this paper solely examines neutral evolution.

These research directions collectively point toward a more comprehensive understanding of how population structure shapes evolutionary timescales. The interplay between spatial organization, mutation, selection, and various metrics of diversity represents a rich space for theoretical exploration with significant practical implications for ecology, virology, cultural evolution, and other fields.

## Methods

Next, we formulate our model and mathematical methods. See the SI Section 1 for further details and proofs.

### Model

We consider a population of *N* individuals undergoing a selection process with drift. The population structure is represented by an unweighted graph of *N* vertices (nodes). Individuals in the population reside on the vertices of the graph. The edges between individuals can be either bidirectional (two-way) or unidirectional (one-way); if all edges are bidirectional, we refer to the graph representing the population structure as undirected. Initially, each individual is a unique type. At each step of the evolutionary process, an individual is selected for birth and an individual is selected for death. The individual selected for death is removed from the population. The individual selected for birth places a copy of itself at the location of the individual selected for death. Each state of the process can be represented by a vector $x\equiv (x_{1},\ldots ,x_{N})$, where $x_{i}\in \{1,\ldots ,N\}$ indicates the type residing at location *i* on the graph.

### Dynamics details

We consider two different dynamics on the population.

- **bd updating:** Under bd dynamics, individual *i* is chosen for birth uniformly at random from the population to give birth. Then individual *j* is chosen for death uniformly at random from the outgoing neighbors of *i*.
- **db updating:** Under db dynamics, individual *j* is chosen for death uniformly at random from the population to give birth. Then individual *i* is chosen for birth uniformly at random from the incoming neighbors of *j*.

We note that bd and db are typically stylized as bd and db in the literature of evolutionary dynamics (13, 18). The capitalized letter in “Birth” (“Death”) signifies that the individual selected for birth (death) is chosen proportional to its fitness, whereas the individual selected for death (birth) is chosen uniformly at random. Our evolutionary dynamics model corresponds to neutral evolution and thus all individuals have the same fitness. Therefore we do not capitalize any letters in the names of the updating rules.

### Absorption time

The process always reaches a homogeneous state where there is only one type in the population. From a homogeneous state, no more state changes can occur. These homogeneous states are the only absorbing states in the Markov chain describing the process. The absorption time is the number of steps until an absorbing state is first reached. The expected absorption times starting in state x, denoted $\tau_{x}$, are the solution to the system of linear equations


(15) $$\tau_{x}=\left\{ \begin{array}{cc} 0 & \text{if}\;x_{1}=\cdots =x_{N},\\ 1+\sum_{x^{\prime }}p_{x\to x^{\prime }}\cdot \tau_{x^{\prime }}\; & \text{otherwise} \end{array}\right.$$

The expression $p_{x\to x^{\prime }}$ is the probability of transitioning to state $x^{\prime }$ in the next step given the current state is x. We are interested in the expected absorption time for x=(1,…,N). In general, Eq. 15 has exponential size and becomes intractable to solve for large *N*.

### Fixation probability

Fixation of type *i* occurs when all individuals in the population are of type *i*. We note that since the process has no mutation, a type that has taken over the population will remain fixated indefinitely. The fixation probability of type *i* is the probability that type *i* fixates. Fixation probabilities depend on the initial configuration of types and the governing dynamics.

### Graph families

We examine properties of various graph families. Each graph in a graph family is indexed by its size *N*. Well-mixed populations are represented by a complete graph with self-loops. The cycle graph family consists of undirected graphs where each vertex is connected to exactly two other vertices, forming a single closed loop. The star graph family consists of undirected graphs with a central vertex that is directly connected to all other vertices; these peripheral vertices have no connections to each other. The double star graph family consists of graph formed by joining two stars graph of the same size by connected their centers; if *N* is odd, the star sizes differ by one. An undirected graph is regular if each vertex has the same number of neighbors. Complete and cycle graph families consists of regular graphs whereas star and double star graph families contain graphs that are not regular. A contracting star is a directed graph consisting of a central vertex connected to multiple blades of the same size. Each blade is made by taking a bidirectional path and, for each vertex, adding directed edges to vertices on its left. Then, the left most vertex of the blade is connected to the center vertex bidirectionally.

### Mass increment method

Frequently in our mathematical analysis of expected absorption times, we argue that some “potential” function of the population state increases overtime by at least a nonnegligible constant rate in expectation. We choose a potential function such that it is bounded and achieves its extreme values only when the population is homogeneous. Thus we can compute an upper bound for the expected absorption time since the potential function will reach its extreme eventually. Similarly, we can track the variance of a potential function overtime. Observing the variance in the potential function allows us to compute a lower bound on the expected absorption time.

### Two types to *N* types

Previous work on absorption times has concentrated on dynamics when only two types are present in the population. Our method of analyzing the case of *N* types involves examining the process with two types and then concluding that starting with *N* types does not significantly slow down the process. The logic is as follows: Assign each type to one of two “meta”-types such that the original types are roughly split between the two meta-types. Then we run the process until one of the meta-types has fixated. At the end of this phase there must be roughly half of the number of original types remaining. We recursively repeat this meta-type assignment with the remaining individuals. Since the number of types is reduced by a half after each phase, the number of phases is logarithmic in the population size.

### Diversity

We measure diversity by the number of distinct types in the population. A population with *N* types has maximum diversity and a population with one type has no diversity.

### Characteristic curves

At each time step of the evolutionary process, we can compute the diversity of the population. Diversity can only decrease overtime since no new types arise in the population. The characteristic curve of a population structure maps time to the expected diversity at that time in the population.

### Properties of small graphs

Using the nauty software suite, we calculated the exact expected absorption times for all undirected graphs of sizes N≤8 under bd and db updating (53). We created a linear system similar to Eq. 15 but we removed symmetries. Given that location *i* is occupied by one of *N* types at any given time, there are $N^{N}$ possible states x in Eq. 15; for N=8 there are $8^{8}\approx 1.6\times 10^{7}$ possible states. However, each type has the same relative fitness and does not mutate. Thus an unlabeled partition of the population based on the locations of the types suffices to create a system for the absorption time. Each state of our reduced system corresponds to a partition of {1,…,N}. Thus, the number of equations in our system is the *N*th Bell number; the 8th Bell number is 4140. For *Ω*, a partition of {1,…,N}, our reduced system is


(16) $$\tau_{\Omega }=\left\{ \begin{array}{cc} 0 & \text{if}\;|\Omega |=1,\\ 1+\sum_{\Omega^{\prime }}p_{\Omega \to \Omega^{\prime }}\cdot \tau_{\Omega^{\prime }}\; & \text{otherwise} \end{array}\right.$$

### Properties of large graphs

We conducted simulations of both bd and db updating to estimate expected absorption times. We examined graphs of sizes up to N=100. For undirected graphs we estimated the expected absorption times for complete, cycle, double star, and star graphs. We conducted numerical calculations and simulations on a high-performance remote computing cluster and across multiple nodes in order to speed up our data collection.

## Supplementary Material

pgaf252_Supplementary_Data

## Data Availability

All simulations and numerical calculations were performed using Python 3.11. Our code is available at https://github.com/harvard-evolutionary-dynamics/diversity-time/ and 10.5281/zenodo.14673093.

## Preprints

This manuscript was posted on a preprint server: https://arxiv.org/abs/2503.09841.

## References

[1] Nowak MA, May RM. 1992. Evolutionary games and spatial chaos. Nature. 359(6398):826–829.

[2] Nowak MA, Michor F, Iwasa Y. 2003. The linear process of somatic evolution. Proc Natl Acad Sci U S A. 100(25):14966–14969. 14657359 10.1073/pnas.2535419100 PMC299861

[3] Lieberman E, Hauert C, Nowak MA. 2005. Evolutionary dynamics on graphs. Nature. 433(7023):312–316. 15662424 10.1038/nature03204

[4] Nowak MA . Evolutionary dynamics: exploring the equations of life. Harvard University Press, 2006.

[5] Ohtsuki H, Hauert C, Lieberman E, Nowak MA. 2006. A simple rule for the evolution of cooperation on graphs and social networks. Nature. 441(7092):502–505. 16724065 10.1038/nature04605 PMC2430087

[6] Tarnita CE, Antal T, Ohtsuki H, Nowak MA. 2009. Evolutionary dynamics in set structured populations. Proc Natl Acad Sci U S A. 106(21):8601–8604. 19433793 10.1073/pnas.0903019106 PMC2689033

[7] Allen B, Nowak MA. 2012. Evolutionary shift dynamics on a cycle. J Theor Biol. 311(21):28–39. 22814475 10.1016/j.jtbi.2012.07.006 PMC3434466

[8] Allen B, Gore J, Nowak MA. 2013. Spatial dilemmas of diffusible public goods. Elife. 2:e01169. 24347543 10.7554/eLife.01169 PMC3865686

[9] Díaz J, Mitsche D. 2021. A survey of the modified Moran process and evolutionary graph theory. Comput Sci Rev. 39(1):100347.

[10] Adlam B, Chatterjee K, Nowak MA. 2015. Amplifiers of selection. Proc R Soc A Math Phys Eng Sci. 471(2181):20150114.

[11] Pavlogiannis A, Tkadlec J, Chatterjee K, Nowak MA. 2017. Amplification on undirected population structures: comets beat stars. Sci Rep. 7(1):82. 28250441 10.1038/s41598-017-00107-w PMC5427850

[12] Pavlogiannis A, Tkadlec J, Chatterjee K, Nowak MA. 2018. Construction of arbitrarily strong amplifiers of natural selection using evolutionary graph theory. Commun Biol. 1(1):71. 30271952 10.1038/s42003-018-0078-7 PMC6123726

[13] Tkadlec J, Pavlogiannis A, Chatterjee K, Nowak MA. 2020. Limits on amplifiers of natural selection under death-birth updating. PLoS Comput Biol. 16(1):e1007494. 31951609 10.1371/journal.pcbi.1007494 PMC6968837

[14] Allen B, et al 2021. Fixation probabilities in graph-structured populations under weak selection. PLoS Comput Biol. 17(2):e1008695. 33529219 10.1371/journal.pcbi.1008695 PMC7880501

[15] Tkadlec J, Pavlogiannis A, Chatterjee K, Nowak MA. 2021. Fast and strong amplifiers of natural selection. Nat Commun. 12(1):4009. 34188036 10.1038/s41467-021-24271-w PMC8242091

[16] Abbara A, Pagani L, García-Pareja C, Bitbol A-F. 2024. Mutant fate in spatially structured populations on graphs: connecting models to experiments. PLoS Comput Biol. 20(9):e1012424. 39241045 10.1371/journal.pcbi.1012424 PMC11410244

[17] Fruet C, Müller EL, Loverdo C, Bitbol A-F. 2025. Spatial structure facilitates evolutionary rescue by cost-free drug resistance. *PLoS Comput Biol*. 21(4):e1012861. 10.1371/journal.pcbi.1012861 PMC11967957 40179127

[18] Svoboda J, Joshi S, Tkadlec J, Chatterjee K. 2024. Amplifiers of selection for the Moran process with both birth-death and death-birth updating. PLoS Comput Biol. 20(3):e1012008. 38551989 10.1371/journal.pcbi.1012008 PMC11006194

[19] Kopfová L, Tkadlec J. 2025. Colonization times in Moran process on graphs. PLoS Comput Biol. 21(5):e1012868. 40324007 10.1371/journal.pcbi.1012868 PMC12052132

[20] Kuo YP, Carja O. 2024. Evolutionary graph theory beyond pairwise interactions: higher-order network motifs shape times to fixation in structured populations. PLoS Comput Biol. 20(3):e1011905. 38489353 10.1371/journal.pcbi.1011905 PMC10971782

[21] Kuo YP, Carja O. 2024. Evolutionary graph theory beyond single mutation dynamics: on how network-structured populations cross fitness landscapes. Genetics. 227(2):iyae055. 38639307 10.1093/genetics/iyae055 PMC11151934

[22] Tkadlec J, Pavlogiannis A, Chatterjee K, Nowak MA. 2019. Population structure determines the tradeoff between fixation probability and fixation time. Commun Biol. 2(1):138. 31044163 10.1038/s42003-019-0373-y PMC6478818

[23] Bhaumik J, Masuda N. 2024. Constant-selection evolutionary dynamics on weighted networks. *Proc R Soc A*. 480(2296):20240223.

[24] Adlam B, Nowak MA. 2014. Universality of fixation probabilities in randomly structured populations. Sci Rep. 4(1):6692. 25346111 10.1038/srep06692 PMC4209402

[25] Díaz J, Goldberg LA, Richerby D, Serna M. 2016. Absorption time of the Moran process. Random Struct Algo. 49(1):137–159.

[26] Brewster DA, Nowak MA, Tkadlec J. 2024. Fixation times on directed graphs. PLoS Comput Biol. 20(7):e1012299. 39024375 10.1371/journal.pcbi.1012299 PMC11288448

[27] Iwamasa Y, Masuda N. 2014. Networks maximizing the consensus time of voter models. Phys Rev E. 90(1):012816. 10.1103/PhysRevE.90.012816 25122351

[28] McAvoy A, Adlam B, Allen B, Nowak MA. 2018. Stationary frequencies and mixing times for neutral drift processes with spatial structure. Proc R Soc A Math Phys Eng Sci. 474(2218):20180238.

[29] Gao S, Liu Y, Wu B. 2024. The speed of neutral evolution on graphs. J R Soc Interface. 21(214):20230594. 38835245 10.1098/rsif.2023.0594 PMC11346635

[30] Kaveh K, McAvoy A, Nowak MA. 2019. Environmental fitness heterogeneity in the Moran process. R Soc Open Sci. 6(1):181661. 30800394 10.1098/rsos.181661 PMC6366185

[31] Kaveh K, McAvoy A, Chatterjee K, Nowak MA. 2020. The Moran process on 2-chromatic graphs. PLoS Comput Biol. 16(11):e1008402. 33151935 10.1371/journal.pcbi.1008402 PMC7671562

[32] Nowak MA, May RM. 1993. The spatial dilemmas of evolution. Int J Bifur Chaos. 03(01):35–78.

[33] Nakamaru M, Matsuda H, Iwasa Y. 1997. The evolution of cooperation in a lattice-structured population. J Theor Biol. 184(1):65–81. 9039401 10.1006/jtbi.1996.0243

[34] Hauert C, Doebeli M. 2004. Spatial structure often inhibits the evolution of cooperation in the snowdrift game. Nature. 428(6983):643–646. 15074318 10.1038/nature02360

[35] Nowak MA . 2006. Five rules for the evolution of cooperation. Science. 314(5805):1560–1563. 17158317 10.1126/science.1133755 PMC3279745

[36] Ohtsuki H, Nowak MA. 2006. Evolutionary games on cycles. Proc R Soc Lond B Biol Sci. 273(1598):2249–2256. 10.1098/rspb.2006.3576 PMC1635521 16901846

[37] Taylor PD, Day T, Wild G. 2007. Evolution of cooperation in a finite homogeneous graph. Nature. 447(7143):469–472. 17522682 10.1038/nature05784

[38] Ohtsuki H, Nowak MA. 2008. Evolutionary stability on graphs. J Theor Biol. 251(4):698–707. 18295801 10.1016/j.jtbi.2008.01.005 PMC2430060

[39] Nathanson CG, Tarnita CE, Nowak MA. 2009. Calculating evolutionary dynamics in structured populations. PLoS Comput Biol. 5(12):e1000615. 20019806 10.1371/journal.pcbi.1000615 PMC2787627

[40] Tarnita CE, Ohtsuki H, Antal T, Fu F, Nowak MA. 2009. Strategy selection in structured populations. J Theor Biol. 259(3):570–581. 19358858 10.1016/j.jtbi.2009.03.035 PMC2710410

[41] Fu F, Nowak MA, Hauert C. 2010. Invasion and expansion of cooperators in lattice populations: prisoner’s dilemma vs. snowdrift games. J Theor Biol. 266(3):358–366. 20619271 10.1016/j.jtbi.2010.06.042 PMC2927800

[42] Van Veelen M, García J, Rand DG, Nowak MA. 2012. Direct reciprocity in structured populations. Proc Natl Acad Sci U S A. 109(25):9929–9934. 22665767 10.1073/pnas.1206694109 PMC3382515

[43] Allen B, Nowak MA. 2014. Games on graphs. EMS Surv Math Sci. 1(1):113–151.

[44] Allen B, et al 2017. Evolutionary dynamics on any population structure. Nature. 544(7649):227–230. 28355181 10.1038/nature21723

[45] Donnelly P, Welsh D. Finite particle systems and infection models. In: *Mathematical Proceedings of the Cambridge Philosophical Society*. Vol. 94, Cambridge University Press, 1983. p. 167–182.

[46] Nordborg M, Krone SM. Separation of time scales and convergence to the coalescent in structured populations. In: *Modern developments in theoretical population genetics: the legacy of gustave malécot*. Oxford University Press, 2002. p. 194–232.

[47] Nordborg M . Coalescent theory. In: *Handbook of Statistical Genomics: Two Volume Set*. John Wiley & Sons, Ltd., 2019. p. 145–30.

[48] Allen B, McAvoy A. 2024. The coalescent in finite populations with arbitrary, fixed structure. Theor Popul Biol. 158(208):150–169. 38880430 10.1016/j.tpb.2024.06.004

[49] Arora S, Barak B. Computational complexity: a modern approach. Cambridge University Press, 2009.

[50] Broom M, Hadjichrysanthou C, Rychtář J. 2010. Evolutionary games on graphs and the speed of the evolutionary process. Proc R Soc A Math Phys Eng Sci. 466(2117):1327–1346.

[51] Goldberg LA, Roth M, Schwarz T. 2024. Parameterised approximation of the fixation probability of the dominant mutation in the multi-type Moran process. Theor Comput Sci. 1016(11):114785.

[52] Cooper C, Rivera N. The linear voting model. In: *43rd International Colloquium on Automata, Languages, and Programming (ICALP 2016)*. Schloss Dagstuhl-Leibniz-Zentrum fuer Informatik, 2016.

[53] McKay BD, Piperno A. 2014. Practical graph isomorphism, II. J Symb Comput. 60(3):94–112.

