# Supplementary material for: Maintaining diversity in structured populations
Source: PNAS Nexus. 2025 Aug 8;4(8):pgaf252. doi: 10.1093/pnasnexus/pgaf252 (PMC12363668; doi:10.1093/pnasnexus/pgaf252)
Supplement: pgaf252_Supplementary_Data [file pgaf252_supplementary_data.pdf]

Supplementary Information: Maintaining diversity in structured populations

Contents

1 Model

We consider the multi-type Moran process [1]. Let $G = (V, E)$ be a simple connected graph with vertex set $V \equiv \{1, \ldots, N\}$ and edge set E. Unless otherwise stated, graphs are undirected, connected, and have neither self-loops nor multiple edges between the same nodes. Let $\Gamma\colon V \to 2^V$ be such that $\Gamma(u) = \{v \mid (u, v) \in E\}$ for each $u \in V$. For a set of types τ, let $f\colon \tau \to \mathbb{Q}_{\geqslant 1}$ be a fitness function. Let Ω be the set of all functions that maps elements of V to elements of τ. In other words, Ω is the set of all possible *configurations of types* on the vertices of G. We sometimes refer to an element of Ω as a *configuration* for brevity. With respect to G, τ, f, and $X_0 \in \Omega$, the *multi-type Moran process* is a discrete-time stochastic process $(X_t)_{t \geqslant 0}$ such that for each $t = 0, 1, 2, \ldots$,

1. $X_{t+1} \in \Omega$, and
2. X_{t+1} is such that $X_{t+1}(D_{t+1}) = X_t(B_{t+1})$ and $X_{t+1}(u) = X_t(u)$ for all $u \in V \setminus \{D_{t+1}\}$; denote this operation $R(X_t, D_{t+1}, B_{t+1}) = X_{t+1}$

where for a Birth-death process, we have

1. $B_{t+1} = u$ with probability $f(X_t(u))/\sum_{x \in V} f(X_t(x))$ for each $u \in V$, and
2. $D_{t+1} = v$ with probability $1/\deg(B_{t+1})$ for each $v \in \Gamma(B_{t+1})$.

and for the death-Birth process, we have

1. $D_{t+1} = v$ with probability $1/|V|$ for each $v \in V$, and
2. $B_{t+1} = u$ with probability $f(X_t(u))/\sum_{x \in \Gamma(D_{t+1})} f(X_t(x))$ for each $u \in \Gamma(D_{t+1})$.

In this work, we will consider constant fitness functions unless otherwise stated. When the fitness function is constant, we will denote Birth-death (Bd) and death-Birth (dB) processes as birth-death (bd) and death-birth (db) processes, respectively. We will also only consider the case where both $|\tau| = |V|$ and $X_0(V) = \tau$ unless otherwise stated. For a graph $G = (V, E)$ and $U \subseteq V$, let $\phi_G(U) := \sum_{u \in U} \frac{1}{\deg(u)}$ where $\deg\colon V \to \mathbb{Z}_{\geqslant 1}$ is the degree of u in G. [2].

Consider a connected undirected graph where initially every vertex is occupied by a distinct type. The *diversity time* for a particular update rule is the expected number of steps the process takes until only one type remains. We call a step of the process *active* if the placements of the wild-types or mutants have changed as a result of this step.

2 Well-mixed

Consider running the multi-type process on a complete graph with self-loops and n vertices; this is known as a well-mixed population. Due to the symmetries of a complete graph, we can track each state of this multi-type process by keeping track of solely the abundance of each type. We use common notation in combinatorics to track such abundances; see [3].

Let $\lambda \equiv (\lambda_1, \ldots, \lambda_\ell)$ such that $\lambda_i \in \mathbb{Z}_{>0}$ for each $i \in [\ell]$, $\lambda_i \geqslant \lambda_{i+1}$ for each $i \in [\ell - 1]$, and $\sum_{i=1}^{\ell} \lambda_i = n$. Thus λ is an *integer partition of* n of *length* ℓ. We use the notation $\lambda \vdash n$ to denote that λ is an integer partition of n. If $\lambda \vdash n$ is of length ℓ and $i \notin [\ell]$, we let $\lambda_i = 0$.

Notice that this multi-type Moran process is equivalent to the following partition walk.

Definition 1 (Frequency-dependent partition walk). *Given $\lambda \vdash n$ of length ℓ, a step in the walk from λ to $\lambda'' \vdash n$ consists of following operations:*

1. *(birth) Choose $r \in [\ell]$ with probability λ_r/n;*
2. *(death) Choose $r' \in [\ell]$ with probability $\lambda_{r'}/n$;*

3. *(replacement)*
   (a) *Construct a partition λ' by setting $\lambda'_r = \lambda_r - 1$, $\lambda'_{r'} = \lambda_r + 1$, and setting $\lambda'_i = \lambda_i$ for the remaining indices;*
   (b) *Construct a partition λ'' by sorting the values of λ' in decreasing order and then removing any indices of λ' that have value 0;*

   Denote this operation $R(\lambda, r, r') = \lambda''$.

Notice that the singleton partition is the sole absorbing state of this walk. Denote hitting time of the frequency-dependent partition walk from any partition $\lambda \vdash n$ to the singleton partition (n) as t^{hit}_λ.

Theorem 1. *Let $\lambda \vdash n$ have length ℓ. Then*

$$\mathbb{E}\left[t^{hit}_\lambda\right] = n^2 - n - \sum_{i=1}^{\ell}\sum_{k=0}^{\lambda_i - 1} \frac{(n+\lambda_i-2k)k}{n-k}. \tag{1}$$

One intuitive way to think about the formula is this (see Fig. 1): Think about the "histogram" of the partition λ and for each $h \geqslant 0$, denote by b_h the number of boxes above the line $y = h$. (In particular, $b_0 = n$.) An explicit formula for b_h is $\sum_i \max(\lambda_i - h, 0)$. Then

$$\mathbb{E}\left[t^{\text{hit}}_\lambda\right] = n \cdot \left(n - \sum_{h=0}^{n-1} \frac{b_h}{n-h}\right).$$

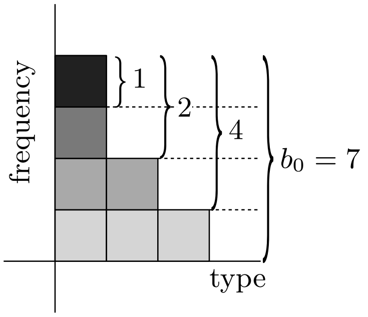

Figure 1: One intuitive way to think about the formula for the complete graph. Here $n = 7$ and $\lambda = (4, 2, 1)$, thus $(b_0, b_1, b_2, b_3) = (7, 4, 2, 1)$. Then $\mathbb{E}\left[t^{\text{hit}}_\lambda\right] = 7 \cdot (7 - \frac{7}{7} - \frac{4}{6} - \frac{2}{5} - \frac{1}{4}) = 32.78\overline{3}$.

We first prove the intuition behind the formula in Fig. 1 and then prove Theorem 1.

Lemma 1. *Let $\lambda \vdash n$ have length ℓ. Then*

$$n^2 - n - \sum_{i=1}^{\ell}\sum_{k=0}^{\lambda_i - 1} \frac{(n+\lambda_i-2k)k}{n-k} = n \cdot \left(n - \sum_{h=0}^{n-1} \frac{b_h}{n-h}\right). \tag{2}$$

Proof. Using the notation $\mathbb{1}[P]$ to denote the indicator function of the predicate P, we interchange summation on the left hand side to get

$$n^2 - n - \sum_{i=1}^{\ell}\sum_{k=0}^{\lambda_i - 1} \frac{(n+\lambda_i-2k)k}{n-k} = n^2 - n - \sum_{k=0}^{n-1}\sum_{i=1}^{\ell} \frac{(n+\lambda_i-2k)k \cdot \mathbb{1}[k < \lambda_i]}{n-k} \tag{3}$$

Notice that $n + \lambda_i - 2k = (n-k) + (\lambda_i - k)$. This means that

$$\begin{aligned}
n^2 - n - \sum_{k=0}^{n-1}\sum_{i=1}^{\ell} \frac{(n+\lambda_i-2k)k \cdot \mathbb{1}[k < \lambda_i]}{n-k} &= n^2 - n - \sum_{k=0}^{n-1}\sum_{i=1}^{\ell} \frac{k(n-k)}{n-k} \cdot \mathbb{1}[k < \lambda_i] - \sum_{k=0}^{n-1}\sum_{i=1}^{\ell} \frac{k(\lambda_i - k)}{n-k} \cdot \mathbb{1}[k < \lambda_i] && (4)\\
&= n^2 - n - \sum_{k=0}^{n-1}\sum_{i=1}^{\ell} k \cdot \mathbb{1}[k < \lambda_i] - \sum_{k=0}^{n-1}\sum_{i=1}^{\ell} \frac{k \cdot \max(\lambda_i - k, 0)}{n-k} && (5)\\
&= n^2 - n - \sum_{k=0}^{n-1} k \sum_{i=1}^{\ell} \mathbb{1}[k < \lambda_i] - \sum_{k=0}^{n-1} \frac{k}{n-k} \sum_{i=1}^{\ell} \max(\lambda_i - k, 0) && (6)\\
&= n^2 - n - \sum_{i=1}^{\ell}\sum_{k=0}^{\lambda_i - 1} k - \sum_{k=0}^{n-1} \frac{k \cdot b_k}{n-k}. && (7)
\end{aligned}$$

Now notice that

$$\sum_{k=0}^{n-1} \frac{k \cdot b_k}{n-k} = \sum_{k=0}^{n-1} \frac{n \cdot b_k}{n-k} - \sum_{k=0}^{n-1} \frac{(n-k) \cdot b_k}{n-k} \tag{8}$$

$$= n \cdot \sum_{k=0}^{n-1} \frac{b_k}{n-k} - \sum_{k=0}^{n-1} b_k. \tag{9}$$

We also have that

$$\sum_{k=0}^{n-1} b_k = \sum_{k=0}^{n-1} \sum_{i=1}^{\ell} \max(\lambda_i - k, 0) = \sum_{k=0}^{n-1} \sum_{i=1}^{\ell} \sum_{h=k}^{\lambda_i - 1} 1 = \sum_{i=1}^{\ell} \sum_{k=0}^{\lambda_i - 1} \lambda_i - k. \tag{10}$$

Thus Equation (7) becomes

$$n^2 - n - \sum_{i=1}^{\ell} \sum_{k=0}^{\lambda_i - 1} k - \sum_{k=0}^{n-1} \frac{k \cdot b_k}{n-k} = n^2 - n - \left(\sum_{i=1}^{\ell} \sum_{k=0}^{\lambda_i - 1} k \right) - \left(n \cdot \sum_{k=0}^{n-1} \frac{b_k}{n-k} - \sum_{i=1}^{\ell} \sum_{k=0}^{\lambda_i - 1} \lambda_i - k \right) \tag{11}$$

$$= n^2 - n - 2 \cdot \left(\sum_{i=1}^{\ell} \sum_{k=0}^{\lambda_i - 1} k \right) + \left(\sum_{i=1}^{\ell} \sum_{k=0}^{\lambda_i - 1} \lambda_i \right) - \left(n \cdot \sum_{k=0}^{n-1} \frac{b_k}{n-k} \right) \tag{12}$$

$$= n^2 - n - \left(\sum_{i=1}^{\ell} \lambda_i \cdot (\lambda_i - 1) \right) + \left(\sum_{i=1}^{\ell} \lambda_i^2 \right) - \left(n \cdot \sum_{k=0}^{n-1} \frac{b_k}{n-k} \right) \tag{13}$$

$$= n^2 - n + \left(\sum_{i=1}^{\ell} \lambda_i \right) - \left(n \cdot \sum_{k=0}^{n-1} \frac{b_k}{n-k} \right) \tag{14}$$

$$= n^2 - n + (n) - \left(n \cdot \sum_{k=0}^{n-1} \frac{b_k}{n-k} \right) \tag{15}$$

$$= n^2 - \left(n \cdot \sum_{k=0}^{n-1} \frac{b_k}{n-k} \right) \tag{16}$$

$$= n \cdot \left(n - \sum_{k=0}^{n-1} \frac{b_k}{n-k} \right). \tag{17}$$

□

Now we prove Theorem 1.

Proof of Theorem 1. We encode the problem as a Markov chain. The states are the partitions of n; for any partitions $\lambda, \mu \vdash n$, the transition probability from λ to μ is $\sum_r \sum_{r'} \lambda_r \lambda_{r'} / n^2$ where the sum is over all r, r' such that $R(\lambda, r, r') = \mu$.

For an absorbing Markov chain with states $\{s_0, \ldots, s_m\}$ where s_0 is the only absorbing state, and transition probabilities (q_{ij}), the expected times to absorption $h_0, \ldots, h_m$ are the unique solution to the following system of linear equations:

$$h_i = \begin{cases} 0 & \text{if } i = 0, \\ 1 + \sum_{j=0}^{m} q_{ij} h_j & \text{otherwise.} \end{cases}$$

Thus $(\mathbb{E}\left[t_\lambda^{\text{hit}}\right])_{\lambda \vdash n}$ is the unique solution $h((\lambda))_{\lambda \vdash n}$ to the system of linear equations:

$$h(\lambda) = \begin{cases} 0 & \text{if } \lambda = (n), \\ 1 + \sum_r \sum_{r'} \frac{\lambda_r \lambda_{r'}}{n^2} \cdot h(R(\lambda, r, r')) & \text{otherwise.} \end{cases}$$

First, to show Eq. (1) holds, we consider when $\lambda = (n)$. Then we have $h(\lambda) = 0$ and

$$n^2 - n - \sum_{k=0}^{n-1} \frac{(n + n - 2k)k}{n-k} = n^2 - n - \sum_{k=0}^{n-1} 2k = n^2 - n - (n^2 - n) = 0.$$

Next, consider the case when $\lambda \neq (n)$. Denote $T(m) := \sum_{k=1}^{m-1} \frac{(n+m-2k)k}{n-k}$ and $g(\lambda) = n^2 - n - \sum_{i=1}^{\ell} T(\lambda_i)$. We want to show $h(\lambda) = g(\lambda)$ for each $\lambda \neq (n)$. Further, let $c_j^\lambda := \#\{i \in [\ell] : \lambda_i = j\}$. Thus $\sum_{i=1}^{\ell} T(\lambda_i) = \sum_{j=1}^{n} c_j^\lambda \cdot T(j)$. Now for any $r \neq r'$,

by plugging in $g(\lambda)$ into $h(\lambda)$ for each $\lambda \vdash n$, we can write

$$\begin{aligned} g(R(\lambda, r, r')) &= \left(n^2 - n - \sum_{j=1}^{n} c_j \cdot T(j)\right) - (T(\lambda_r) + T(\lambda_{r'})) + (T(\lambda_r - 1) + T(\lambda_{r'} + 1)) \\ &= g(\lambda) - (T(\lambda_r) + T(\lambda_{r'})) + (T(\lambda_r - 1) + T(\lambda_{r'} + 1)) . \end{aligned}$$

When $r = r'$, we have $g(R(\lambda, r, r')) = g(\lambda)$. Hence, we need to show

$$\sum_{r} \sum_{r' \neq r} \frac{\lambda_r \lambda_{r'}}{n^2} (-T(\lambda_r) - T(\lambda_{r'}) + T(\lambda_r - 1) + T(\lambda_{r'} + 1)) = 1. \tag{18}$$

First, we have

$$\begin{aligned} T(m+1) - T(m) &= \sum_{k=1}^{m} \frac{(n+m-2k+1)k}{n-k} - \sum_{k=1}^{m-1} \frac{(n+m-2k)k}{n-k} \\ &= \frac{(n-m+1)m}{n-m} + \sum_{k=1}^{m-1} \frac{(n+m-2k+1)k}{n-k} - \frac{(n+m-2k)k}{n-k} \\ &= \frac{(n-m+1)m}{n-m} + \sum_{k=1}^{m-1} \frac{k}{n-k} \\ &= m + \frac{m}{n-m} + \sum_{k=1}^{m-1} \frac{k}{n-k} \\ &= m + \sum_{k=1}^{m} \frac{k}{n-k}. \end{aligned}$$

Let $U(m) := \sum_{k=1}^{m-1} \frac{k}{n-k}$. Thus the left hand side of Eq. (18) is

$$\begin{aligned} &\sum_{r} \sum_{r' \neq r} \frac{\lambda_r \lambda_{r'}}{n^2} (U(\lambda_{r'} + 1) - U(\lambda_r) + \lambda_{r'} - \lambda_r + 1) \\ &= \sum_{r} \sum_{r' > r} \frac{\lambda_r \lambda_{r'}}{n^2} (U(\lambda_{r'} + 1) - U(\lambda_r) + \lambda_{r'} - \lambda_r + 1 + U(\lambda_r + 1) - U(\lambda_{r'}) + \lambda_r - \lambda_{r'} + 1) \\ &= \sum_{r} \sum_{r' > r} \frac{\lambda_r \lambda_{r'}}{n^2} \left(1 + \frac{\lambda_r}{n - \lambda_r} + 1 + \frac{\lambda_{r'}}{n - \lambda_{r'}}\right) \\ &= \sum_{r} \sum_{r' > r} \frac{\lambda_r \lambda_{r'}}{n^2} \left(\frac{n}{n - \lambda_r} + \frac{n}{n - \lambda_{r'}}\right) \\ &= \sum_{r} \sum_{r' > r} \frac{\lambda_r \lambda_{r'}}{n} \left(\frac{1}{n - \lambda_r} + \frac{1}{n - \lambda_{r'}}\right) \\ &= \frac{1}{n} \sum_{r} \frac{\lambda_r}{n - \lambda_r} \sum_{r' > r} \lambda_{r'} + \frac{1}{n} \sum_{r} \frac{\lambda_r}{n - \lambda_r} \sum_{r' < r} \lambda_{r'} \\ &= \frac{1}{n} \sum_{r} \frac{\lambda_r}{n - \lambda_r} \sum_{r' > r} \lambda_{r'} + \frac{1}{n} \sum_{r} \frac{\lambda_{r'}}{n - \lambda_{r'}} \sum_{r < r'} \lambda_r \qquad \text{(by relabeling)} \\ &= \frac{1}{n} \sum_{r} \frac{\lambda_r}{n - \lambda_r} \sum_{r' \neq r} \lambda_{r'}. \end{aligned}$$

Finally, we use the fact that $\lambda_1 + \cdots + \lambda_\ell = n$ to obtain

$$\frac{1}{n} \sum_{r} \frac{\lambda_r}{n - \lambda_r} \sum_{r' \neq r} \lambda_{r'} = \frac{1}{n} \sum_{\lambda_r} n - \lambda_r (n - \lambda_r) = \frac{1}{n} \sum_{r} \lambda_r = \frac{1}{n} \cdot n = 1.$$

Therefore $h(\lambda) = g(\lambda)$ for each $\lambda \vdash n$, and thus $\mathbb{E}\left[t_\lambda^{\text{hit}}\right] = g(\lambda)$ for each $\lambda \vdash n$. □

Corollary 1. *Let* $(1, \ldots, 1) \vdash n$*. Then* $\mathbb{E}\left[t_{(1,\ldots,1)}^{hit}\right] = n^2 - n$.

Proof. By Theorem 1,

$$\mathbb{E}\left[t^{\text{hit}}_{(1,\ldots,1)}\right] = n^2 - n - \sum_{i=1}^{n}\sum_{k=0}^{0}\frac{(n+1-2k)k}{n-k} = n^2 - n.$$

□

Corollary 2. *The expected absorption time of the multi-type process on a complete graph with no self-loops and n vertices is $(n-1)^2$ when starting with n different types.*

Proof. Let $G = (V, E + \{(u,u) \mid u \in V\})$ be a complete graph with self-loops on n vertices. At each step, regardless of the configuration, there is a $1 - 1/n$ probability a birth occurs along E. Otherwise, the configuration does not change. Thus by linearity of expectation, the expected absorption time for G is the same as the expected absorption time for $G' = (V, E)$ where each step has weight $(1-1/n)^{-1}$ (expectation of a geometric random variable with success probability $1 - 1/n$). So from Corollary 1 we get that the expected absorption time for G' is

$$(1 - 1/n) \cdot (n^2 - n) = ((n-1)/n) \cdot n(n-1) = (n-1)^2. \tag{19}$$

□

3 Cycles

We consider the multi-type process on a cycle and show the exact expected time from any configuration. The result uses a potential function that tracks the expected absorption time and implies $\Theta(n^3)$ for the diversity time. We present the proof for completeness, the proof can also be derived as a special case of [4].

Observation 1. *Since every type starts with only one individual and reproductions happen only on the boundaries of contiguous segments of a type, each type always occupies a contiguous segment of the cycle.*

Similar to our analysis of well-mixed populations, we use λ, an *integer partition of* n of *length* ℓ. This time, λ does not encode the whole configuration on the graph, there are nonisomorphic configurations that have the same λ. However, λ and Observation 1 are enough to track the expected number of steps.

Theorem 2. *Given λ on a cycle where every type forms a contiguous segment of the cycle, we have*

$$\mathbb{E}\left[t^{hit}_{\lambda}\right] = \frac{(n-1)n(n+1)}{6} - \sum_{h=0}^{n-1} b_h \cdot h,$$

where $b_h := \sum_{i\in[\ell]} \max(0, \lambda_i - h)$.

Proof. Given a configuration λ, we use the potential function

$$\phi(\lambda) = n^2 - n - \sum_{i=1}^{\ell}\sum_{k=0}^{\lambda_i - 1}\frac{(n+\lambda_i-2k)k}{n-k}$$

which is the expected absorption time on a complete graph with n vertices.

We show that given the potential and λ, the expected change is -1 unless the whole cycle is occupied by only one type. Since the potential is 0 if the cycle is occupied by one type, this implies that the potential tracks the expected number of steps.

We can substitute the bd or db process by selecting a random edge and then selecting the direction of reproduction. This change keeps the same replacement probabilities.

We examine the potential change conditioned on the fact that an edge between two different individuals i and j is selected. Suppose that $\lambda_i \geqslant \lambda_j$ and i replaced individual j. Then b_h changes to b'_h and $b'_h - b_h = 1$ for all h from λ_j to λ_i. Similarly, when individual j replaces i, the difference in $b'_h - b_h$ is -1 for all h from $\lambda_j + 1$ to $\lambda_i - 1$.

Since the edge between i and j has the same probability of being selected in both directions, we have the expected change in the potential

$$-\frac{1}{2}\lambda_i - \frac{1}{2}\lambda_j\,.$$

We have this change in potential for any pair of neighboring types. There are $|\ell|$ neighboring types, conditioned that an edge between them is selected, we have the change in potential

$$\frac{1}{|\ell|}\sum_{i\in[\ell]} -\lambda_i = -\frac{n}{|\ell|}\,.$$

Finally, an edge between two individuals is selected with probability $\frac{|\ell|}{n}$, which gives the change in potential -1 in one step. □

4 Paths

The path is created from the cycle by removing a link between vertex v_1 and v_n. We use the potential for the cycle with minor modifications and prove bound $\Theta(n^3)$ for the diversity time.

Theorem 3. *The diversity time on a path is* $\Theta(n^3)$.

Proof. First, we show the lower bound on the diversity time. We show that the expected fixation time is $\Omega(n^3)$ for two types, each occupying a continuous half of the path. This configuration can be simulated by the unbiased one-dimensional Markov Chain where the absorbing states are at distances $\Theta(n)$ from the starting state. The expected time on this Markov Chain is $\Theta(n^2)$ from Proposition 2.1 of [5]; however, we have an n times slowdown because most of the time individuals not on the boundary reproduce.

Observe that this also shows that for two types, the expected time is $\mathcal{O}(n^3)$. In other words, the time until one type disappears is bounded by $\mathcal{O}(n^3)$. This means that the diversity time for a constant number of types is also $\mathcal{O}(n^3)$.

For many types, we use the same potential as in the cycle but we change the process such that in every configuration, the potential decreases by at least -1.

Any type that at any time occupies v_1, v_2 (neighbor of v_1), v_{n-1} (neighbor of v_n), or v_n is treated as being type 0. Type 0 is an amalgamation of at most 4 types, two from each end of the path. In the beginning, type 0 consists of four types, and any type that becomes type 0 needs to claim v_2 or v_{n-1}, but if this happens, there can be only one other type in v_1 or v_n.

When no type is changed to type 0, the potential decreases as in the cycle. Our changes ensure that the type 0 occupies both vertices of degree 1 and their neighbors. So, the only vertices that have different replacement probabilities than the cycle are occupied by type 0, and the replacement of the same type does not change the potential.

When another type becomes type 0, then the potential decreases more than in the cycle, it is equivalent to merging two types.

This means the potential decreases by at least 1 every step. Therefore the expected time until only one type remains is $\mathcal{O}(n^3)$. The remaining type is type 0, which was created by fusing of at most 4 different types. However, we know that in that case, the expected time is at most $\mathcal{O}(n^3)$.

The expected time on the path is at least $\Omega(n^3)$ even for two types, the time until there are only constantly many types requires $\mathcal{O}(n^3)$ steps and finally getting to one type requires $\mathcal{O}(n^3)$ steps. Therefore the expected time on path is $\Theta(n^3)$. □

5 Stars

5.1 bd updating

We use the results from Section 2 to show a cubic bound for stars. We consider a star with n leaves and thus $N = n + 1$ total vertices.

Theorem 4. *The diversity time for a star in bd updating is* $\mathcal{O}(N^3)$.

Proof. Note that every configuration of the bd process on a graph $G = (V, E)$ with $|V| = n$ can be represented by a partition of $\{1, \ldots, n\}$. Let Π be all partitions of the n leaves of a star. We define a potential function $\phi\colon \Pi \to \mathbb{R}$ that maps a given partition λ of $\{1, \ldots, n\}$ to the expected absorption time on a complete graph with n vertices with initial configuration λ.

We will track only the reproductions of individuals on the central vertex and the configuration in the leaves of the star. Any time the center individual reproduces (which happens with probability $1/N$), two things could happen in the previous round:

(A) an individual from a leaf replaced the center individual (with probability $1 - 1/N$), or

(B) the center individual reproduced.

Whenever Situation (A) happens, the probability distribution of the center corresponds to λ up to reordering. Therefore the transition probabilities are the same as in the complete graph: in the previous round, a randomly chosen individual replaced the center. Whenever Situation (B) happens, we examine the probabilities and in some cases, we select the most pessimistic replacement that increases the potential ϕ the most. It is the event where the least prevalent type in λ replaces one individual of the most prevalent type in λ. Even with these changes, we show that, in every configuration, the potential decreases by at least a constant in expectation. This will give the bound $\mathcal{O}(N^2)$ for the number of reproductions of the center from Theorem 7 of [6]. Since the center reproduces with probability $1/N$, counting all reproductions including leaves replacing the center gives the total number of steps $\mathcal{O}(N^3)$.

Now, for the configuration $\lambda \in \Pi$ on the leaves of the star, we have the following potential.

$$\phi(\lambda) = n^2 - n - \sum_{i=1}^{\ell} \sum_{k=0}^{\lambda_i - 1} \frac{(n + \lambda_i - 2k)k}{n - k}.$$

Let $\lambda^{(i)}$ be the configuration of the leaves after i steps of the process. Suppose the center reproduces for the ith step. Then

- with probability $1-1/N$, an individual from a leaf reproduced into the center for the $(i-1)$th step. Then the potential changes by
$$\phi(\lambda^{(i)}) - \phi(\lambda^{(i-1)}) = -1$$
since this is one step in the process for the complete graph with self-loops. The expected absorption time (which is the potential function) will decrease by 1.

- with probability $1/N$, the center individual gave birth for the $(i-1)$th step.
  - with probability $1-1/N$, a leaf individual gave birth for the $(i-2)$th step. We know that $\lambda^{(i-1)}$ and $\lambda^{(i)}$ can only differ in at most two locations. The probability of selecting any of the differing indices is at most $2/n$ since there are n leaves. Observe that the biggest possible change in the potential ϕ is
  $$n-1+\sum_{k=1}^{n-1}\frac{k}{n-k} \leqslant nH_n \leqslant n\log n.$$
  This happens when a type that occupies all but one leaf is replaced. Specifically, for a given configuration λ, the biggest increase in potential happens when the least prevalent type replaces the most prevalent type. Thus with probability at most $2/n$ the potential function has increased by at most $n\log n$ and with remaining probability has decreased by 1.
  - with probability $1/N$, the center individual gave birth for the $(i-2)$th step. By similar logic, the potential can only increase by $n\log n$ at each step.

Putting the pieces together, we get

$$\begin{aligned}
E[\phi(\lambda^{(i)}) - \phi(\lambda^{(i-1)}) \mid \lambda^{(i-1)}] &\leqslant (1-1/N)\cdot(-1) \\
&+ (1/N)\cdot(1-1/N)\,[(2/n)\cdot n\log n + (1-2/n)\cdot(-1)] \\
&+ (1/N)\cdot(1/N)\cdot n\log n \\
&\leqslant -1+1/n \\
&+ (1/n)\cdot(2\log n - 1 + 2/n) \\
&+ (\log n)/n \\
&= -1 + (3\log n)/n + 2/n^2 \\
&= -1 + o(1).
\end{aligned}$$

The completion of the proof follows from the Theorem 7 of [6].

□

5.2 db updating

Theorem 5. *The diversity time on a star in db updating is bounded by $\mathcal{O}(N\log N)$.*

Proof. Let n be the number of leaves of the star and $N=n+1$ be the total number of vertices. We show that with high probability, the individual in the center spreads over the whole graph in n active steps.

Let i be the number of individuals whose types differ from the type of the center individual. Two possibilities exist for an active step:

(A) i decreases, or

(B) the center individual is replaced.

Event (A) happens if one of the individuals different from the center dies; the probability of this event is i/N. Event (B) happens when the center dies (probability $1/N$) and is replaced by a different individual (probability i/n). Thus in one active step, the probability of losing the center is

$$\frac{\frac{1}{N}\frac{i}{n}}{\frac{1}{N}\frac{i}{n}+\frac{i}{N}} = \frac{\frac{1}{n}}{\frac{1}{n}+1} = \frac{1}{N}\,.$$

One type must fixate after at most n active steps if the center was never replaced; this happens with probability

$$\left(1-\frac{1}{N}\right)^n \geqslant 1/e\,.$$

Now, we compute the maximal expected number of all steps until the center fixates or is replaced. Having i different individuals different from the center, the probability of an active step is at least i/N. That means to have all n leaves to be the same type as the center or have the center replaced, we have expected time at most

$$\sum_{i=1}^{n} \frac{N}{i} = N \cdot H_n.$$

Then the total expected time until absorption, T_N, satisfies.

$$T_N \leqslant (N \cdot H_n) + (1 - 1/e) \cdot T_N.$$

Thus $T_N \leqslant eN \cdot H_n \leqslant \mathcal{O}(N \log N)$. □

6 General Lemma for Lower Bounds

In this section, we prove a general lemma about lower bounds for absorption times. We consider a process where one of the types cannot be replaced (is invincible) when occupying certain vertices. Then we show the relationship between this process and the original process. The result is general and holds for bd, db, and all dynamics where the replacement vertices do not depend on the exact neighborhood configurations.

Notation. Let the type a occupy a set of vertices A. We denote the fixation probability of type a occupying the set A by $f(a, A)$. Let T be a random variable that denotes the absorption time of the process when type a initially only occupies A.

The invincible process. Let the *invincible process* for set A be similar to the original process, but where individuals residing on vertices in A cannot be replaced. Let T_A be a random variable that denotes the absorption time of this invincible process for set A where type a initially only occupies A.

Lemma 2. *Let $G = (V, E)$ and $A \subseteq V$. For bd and db updating, we have*

$$E[T] \geqslant \frac{f(a, A)^2}{4} E[T_A] .$$

Proof. We have

$$E[T] = f(a, A)E[T \mid a \text{ fixates}] + (1 - f(a, A))E[T \mid a \text{ becomes extinct}] . \tag{20}$$

Let E_f denote the expected absorption time of the invincible process for A conditioned on the event the trajectory is among the shortest $f(a, A)$-proportion of the possible invincible trajectories. We will first show

$$E[T \mid a \text{ fixates}] \geqslant E_f .$$

Consider running the original process $\mathcal{P}_\emptyset$ and the invincible process $\mathcal{P}_A$ with the same randomness and where type a initially only occupies A. Suppose $\mathcal{P}_\emptyset$ absorbs with type a fixated. Since individuals from A are not replaced, $\mathcal{P}_A$ must have absorbed with type a fixated but in no more steps than $\mathcal{P}_\emptyset$. That means in $\mathcal{P}_A$, type a occupies a superset of vertices at each step compared to the vertices that type a occupies in $\mathcal{P}_\emptyset$. So we have

$$E[T \mid a \text{ fixates}] \geqslant E[T_A \mid a \text{ fixates}] \geqslant E_f.$$

Now, we compare $E[T_A]$ with E_f. We run $\mathcal{P}_A$ for $2E_f$ steps. From Markov's inequality,

$$P\left[T_A > 2E_f \,\middle|\, \mathcal{P}_A \text{ is among shortest } f(a, A)\text{-proportion of invincible trajectories}\right] \leqslant \frac{E_f}{2E_f} = 1/2.$$

Thus

$$P[T_A \leqslant 2E_f] \geqslant f(a, A)/2.$$

So we have that at least $f(a, A)/2$ proportion of invincible process absorbs within $2E_f$ steps. If $\mathcal{P}_A$ does not absorb within $2E_f$ steps, type a occupies vertices from A and possibly some other vertices. From this configuration, the expected time is at most $E[T_A]$, since the set of vertices occupied by a is a superset of A. That means

$$E[T_A] \leqslant 2E_f + \left(1 - \frac{f(a, A)}{2}\right) E[T_A] .$$

That means $E[T_A] \leqslant \frac{4}{f(a,A)} E_f$. Combining the bound on $E[T_A]$ with Equation (20) yields

$$E[T] \geqslant \frac{f(a, A)^2}{4} E[T_A].$$

□

7 Double Stars

In this section, we examine the diversity times on double star in bd updating. Double star has $N = 2n + 2$ vertices and consists of two stars S_n and S'_n where the centers are connected.

We say the configuration is *neutral* if the red type occupies S_n and the blue type occupies S'_n. We say that the center of a star *interacts* with the other star if it first spreads to the center of the other star and then, without being replaced, this center of the star claims a leaf. The interaction happens in two active steps (where we count the active step as a reproduction of the first center and then the successful reproduction or death of the second center).

Lemma 3. *The center of one star interacts with the other star with probability* $\frac{n}{(n+1)^4}$.

Proof. A center is selected with probability $\frac{1}{n+1}$ $(= \frac{2}{2n+2})$ and with probability $\frac{1}{n+1}$ it spreads to the other center. We consider only two possibilities as an active step, either the newly conquered center dies or reproduces to a leaf. The reproduction happens with probability $\frac{1}{n+1} \cdot \frac{n}{n+1}$, otherwise it dies. Multiplying the probabilities gives $\frac{n}{(n+1)^4}$. □

7.1 Lower bound

In the proof, we count only the number of steps in the neutral configuration. It still gives us a bound of $\Omega(N^4)$.

Lemma 4. *The diversity time of a double star under bd updating is* $\Omega(N^4)$.

Proof. We will use Lemma 2 starting in the neutral configuration so that A is the set of vertices on the right star initially occupied by type $a = 2$. In the neutral configuration, both types have the same probability of fixating. Therefore the fixation probability of type 2 in the neutral configuration is $f(a, A) = 1/2$. By Lemma 2, the time until absorption T satisfies

$$E[T] \geqslant f(a, A)^2 E[T_A]/4 = E[T_A]/16 = \Omega(E[T_A]). \tag{21}$$

Thus it suffices to find a time lower bound for the invincible process starting from the neutral configuration. In what follows, we analyze the time in the invincible process.

By Lemma 3, we know that the center of one star interacts with the other star with probability at most $1/n^3$. This means that the expected number of steps until type 2 interacts with the other star starting from the neutral configuration is at least $\Omega(n^3)$. We will show that once type 2 interacts with the other star, type 2 has at most a $\mathcal{O}(1/n)$ probability of fixating. With remaining probability, we return back to the neutral configuration. This means type 2 will need to interact with the other star at least $\Omega(n)$ times in expectation before type 2 fixates. Since the time between tries is $\Omega(n^3)$ in expectation, the total expected time is $\Omega(n^4)$ for the invincible process.

We consider a process on a different graph with fixation probability at most $\mathcal{O}(1/n)$ and show that this is an upper bound for the probability type 2 fixates in the other star in the invincible process between interactions.

Consider a star with $n+1$ vertices where mutant (i.e. type 2) have relative reproductive fitness $1 + 2/n$. We call this the *improved* process. We show that for the improved process, we have a higher ratio between increasing the number of mutants and decreasing the number of mutants as opposed to the invincible process.

For the improved process, the fixation probability with mutants at the center and a leaf is $\mathcal{O}(1/n)$ from [7,8].

Center is occupied. Suppose the center and i leaves belong to type 2 in the invincible process. We have a probability of increasing the number of type 2 individuals $\frac{1}{n+2} \cdot \frac{n-i}{n+1}$. The probability of losing the center is $\frac{n-i}{n+2}$. The ratio is $\frac{1}{n+1}$.

Now, suppose the center and i leaves belong to mutants in the improved process. The probability of increasing the number of mutants is $\frac{1+\frac{2}{n}}{F}\frac{n-i}{n}$, where F is the total fitness of all individuals. The probability of losing the center is $\frac{n-i}{F}$. The ratio is $\frac{1+\frac{2}{n}}{n}$.

Center is not occupied. Suppose i leaves belong to type 2 and the center belongs to type 1 in the invincible process. We have a probability of claiming the center $\frac{i}{n+2} + \frac{1}{n+2} \cdot \frac{1}{n+1}$. The probability of losing a type 2 individual is $\frac{1}{n+2}\frac{i}{n+1}$. The ratio is $n + 1 + \frac{1}{i}$.

Now, suppose i leaves belong to mutants and the center belongs to the residents in the improved process. The probability of claiming the center is $\frac{(1+\frac{2}{n})i}{F}$, where F is the total fitness of all individuals. The probability of losing a mutant is $\frac{1}{F}\frac{i}{n}$. The ratio is $n + 2$.

In both cases, we have a higher probability ratio of type 2 increasing versus decreasing. This is what we wanted to show. □

7.2 Upper bound

Lemma 5. *The diversity time of a double star under bd updating is $\mathcal{O}(N^4)$.*

Proof. First, we prove that all except two types disappear quickly. Then, we argue about the diversity time for only two types.

Multiple types. A double star consists of two n-leaf stars, S_1 and S_2, connected by an edge at their centers, c_1 and c_2, respectively. Suppose we start the process with N types. Consider the vertices of S_1. Using a similar technique to that of Theorem 4, we show that S_1 becomes homogeneous in at most $O(n^3)$ steps in expectation.

Let Π be all partitions of the n leaves of S_1. We define a potential function $\phi\colon \Pi \to \mathbb{R}$ that maps a given partition λ of $\{1, \ldots, n\}$ to the expected absorption time on a complete graph with n vertices with initial configuration λ.

We will track only the reproductions of individuals on the central vertex c_1 and the configuration in the leaves of the star S_1. Any time the center individual at location c_1 reproduces in S_1 (which happens with probability $(1/N) \cdot n/(n+1)$), four things could have happened in the previous round:

(A) an individual from a leaf of S_1 replaced the center individual c_1

(B) the center individual c_1 reproduced onto a leaf of S_1,

(C) the individual at the other center c_2 gave birth onto c_1, or

(D) a death occurred in S_2.

At each time step

- situation (A) has probability $p_A := n/N$ of occurring,
- situation (B) has probability $p_B := (1/N) \cdot n/(n+1)$ of occurring,
- situation (C) has probability $p_C := (1/N) \cdot 1/(n+1)$ of occurring, and
- situation (D) has probability $p_D := 1 - p_A - p_B - p_C = (n/N) + (1/N) \cdot n/(n+1) + (1/N) \cdot 1/(n+1) = 1/2$ of occurring.

Now, for the configuration $\lambda \in \Pi$ on the leaves of the star, we have the following potential.

$$\phi(\lambda) = \mathbb{E}\left[t_\lambda^{\text{hit}}\right] = n^2 - n - \sum_{i=1}^{\ell} \sum_{k=0}^{\lambda_i - 1} \frac{(n + \lambda_i - 2k)k}{n - k}.$$

Similar to the proof of Theorem 4, the largest change in the potential at any step is $n \log n$.

Let $\lambda^{(i)}$ be the configuration of the leaves after i steps of the process. Suppose the center reproduces for the ith step. Then

- with probability p_A, situation (A) occurs for the $(i-1)$th step. Then the potential changes by

  $$\phi(\lambda^{(i)}) - \phi(\lambda^{(i-1)}) = -1$$

  since this is one step in the process for the complete graph with self-loops. The expected absorption time (which is the potential function) will decrease by 1.

- with probability p_B, situation (B) occurs for the $(i-1)$th step.
  - with probability p_A, situation (A) occurs for the $(i-2)$th step. Thus with probability at most $2/n$ the potential function has increased by at most $n \log n$ and with remaining probability has decreased by 1 (see the details of Theorem 4.
  - with probability p_B, situation (B) occurs for the $(i-2)$th step. By similar logic, the potential can only increase by $n \log n$ at each step.
  - with probability p_C, situation (C) occurs for the $(i-2)$th step. Thus potential again can only increased by $n \log n$.
  - with probability p_D, situation (D) occurs for the $(i-2)$th step and there is no change in the potential function.
- with probability p_C, situation (C) occurs in the $(i-1)$th step and the potential can only increase by at most $n \log n$
- with probability p_D, situation (D) occurs in the $(i-1)$th step and the potential does not change.

Putting the pieces together, we get

$$\begin{aligned}E[\phi(\lambda^{(i)}) - \phi(\lambda^{(i-1)}) \mid \lambda^{(i-1)}] &\leqslant p_A \cdot (-1) \\ &+ p_B \cdot [p_A((2/n) \cdot n \log n + (1 - 2/n) \cdot (-1)) + (p_B + p_C) \cdot n \log n + p_D \cdot 0] \\ &+ p_C \cdot n \log n \\ &+ p_D \cdot 0 \\ &\leqslant -p_A + p_A p_B \cdot (2 \log n - 1 + 2/n) + p_B^2 \cdot n \log n + p_C(p_B + 1) \cdot n \log n \\ &= -\frac{n^3}{2(n+1)^3} - \frac{5n^2}{4(n+1)^3} + \frac{5n^2 \log n}{4(n+1)^3} + \frac{n \log n}{2(n+1)^3} \\ &\leqslant -1/2 + o(1).\end{aligned}$$

From the Theorem 7 of [6], S_1 becomes homogeneous in $O(N^3)$ steps in expectation.

Let T_{S_1} (T_{S_2}) be the time it takes until S_1 (S_2) is homogeneous starting with S_1 (S_2) as heterogeneous. From above we have that $E[T_{S_1}] = O(N^3)$. By Markov's inequality, there exist some constants $c > 0$ and $d \in (0, 1)$ such that

$$P(T_{S_1} \geqslant cN^3) \leqslant E[T_{S_1}]/(cN^3) \leqslant d. \tag{22}$$

Let T_2 be the time it takes until S_1 and S_2 are homogeneous starting with S_1 as heterogeneous. Let T' be the time it takes until S_2 is homogeneous given that S_1 is homogeneous. Then for indicator random variable $I\{\cdot\}$ we have the system

$$\begin{aligned}T_2 &\leqslant T_{S_1} + T' \\ T' &\leqslant I\{T_{S_2} < cN^3\} \cdot T_{S_2} + I\{T_{S_2} \geqslant cN^3\} \cdot T_2.\end{aligned}$$

Taking expectations yields the system

$$\begin{aligned}E[T_2] &\leqslant O(N^3) + E[T'] \\ E[T'] &\leqslant 1 \cdot cN^3 + d \cdot E[T_2].\end{aligned}$$

This implies that $E[T_2] \leqslant O(N^3)$.

Two types. Having two types (type 1 and type 2) in the neutral configuration, we compute the expected time. From any configuration with more than two types, we reach the neutral configuration in $\mathcal{O}(n^3)$ steps with constant probability. This result follows from the previous part of the proof for multiple types.

Without loss of generality, suppose that type 2 (on star S_2) invades a leaf of the star that type 1 occupies (S_1). This happens with probability

$$p := (1/N)(1/(n+1)) \cdot (1/N) \cdot (n/(n+1)) = \Theta(1/n^3)$$

in two steps.

Suppose no reproduction from c_1 to c_2 happens in $1/p = \mathcal{O}(n^3)$ steps. This event occurs with constant positive probability q by Markov's inequality. Thus S_1 is invaded without S_2 being invaded. The invader of S_1 has probability at least $\Omega(1/n)$ of conquering S_1. If one or both stars are invaded, in $\mathcal{O}(n^3)$ steps on average, fixation or the neutral configuration is reached. But the neutral configuration is reached with probability $p \cdot \Omega(1/N) = \Omega(1/n^4)$ (S_1 invades S_2 and succeeds while S_1 is being invaded). This implies that the number of visits of all configurations that are not neutral configuration is at most $\mathcal{O}(n^4)$. Moreover, the number of steps until at least one star is invaded from the neutral configuration is $\mathcal{O}(n^3)$ in expectation. This is because from Lemma 3, the probability of spreading between stars is at least $\Omega(1/n^3)$.

With probability $1 - q$, we restart our analysis with $O(n^3)$ steps taken. This gives a recurrence for the expected time T until absorption of

$$T \leqslant (1 - q)(T + \mathcal{O}(n^3)) + q \cdot \mathcal{O}(n^4)$$

which has a solution of $T \leqslant \mathcal{O}(N^4)$

□

8 Barbells

8.1 db lower bound on the highest time

A *barbell* graph with $N = 3n$ vertices consists of a left clique with n vertices, a path with n vertices, and a right clique with n. We assume n is even for simplicity. One end of the path is connected to one vertex in the left clique. The other end of the path is connected to one vertex in the right clique.

Theorem 6. *In the barbell graph, the diversity time is* $\Omega(N^4)$.

Proof. We decompose a barbell graph on $N = 3n$ vertices as cliques K_L and K_R that each have a path P_L (resp. P_R) of length $n/2$ connected to one of its nodes by an edge. Then P_L and P_R are connected by an edge at the endpoints of each of the paths (i.e. at the vertices that have unity degree). A configuration of types on a barbell graph is called *neutral* if one type (type 1) occupies $V(K_L) \cup V(P_L)$ and another type (type 2) occupies $V(K_R) \cup V(P_R)$. We prove a lower bound for when the process starts in a neutral configuration. We use Lemma 2 from this configuration to prove the lower bound we desire. We will use $A = V(K_R) \cup V(P_R)$ as the invincible set of vertices and set type $a = 2$. We have that the fixation probability of type $a = 2$ is $f(a, A) = 1/2$ by symmetry. Therefore by Lemma 2,

$$E[T] \geqslant f(a, A)^2 E[T_A]/4 = E[T_A]/16 = \Omega(E[T_A]).$$

Therefore it suffices to find a time lower bound for the invincible process starting from the neutral configuration. Below, we will analyze the time in the invincible process.

We define four (out of many) possible configurations with two types:

(I) type 1 occupies exactly $V(K_L) \cup V(P_L)$,

(II) type 1 occupies exactly $V(K_L)$,

(III) type 1 occupies a proper non-empty subset of $V(K_L)$, and

(IV) type 1 does not occupy any vertices.

In order for the invincible process to absorb, we must pass through each of the above four configurations at least once. Let T_I, T_{II}, and T_{III} be random variables for the number of steps it takes to reach configuration (IV) in the invincible process starting at the corresponding configurations.

Firstly, we have

$$E[T_I] \geqslant \Omega(N^3) + E[T_{II}]$$

from Theorem 3. We denote the vertex of K_L connected to P_L by and edge as the *bridgehead*.

In configuration (II), with high probability $(1 - 2/N)$ the configuration does not change since either the bridgehead or the node in P_L connected to the bridgehead needs to be selected for death for the configuration to change. Losing the bridgehead to type 2 happens with probability at least $\Omega(1/N^2)$ since this could happen if immediately, the bridgehead is selected for death and the node in P_L connected to the bridgehead is selected for birth. This is only two steps of the process. On the other hand, it is possible to back to configuration (I): the node in P_L connected to the bridgehead is selected for death immediately and then the bridgehead is selected for birth. Then there is at least an $\Omega(1/N)$ chance by analyzing the fixation probability of an invader placed at the end of a path. This would take at least $\Omega(N)$ steps to achieve. Thus

$$E[T_{II}] \geqslant \Omega(1) + (1 - 2/N) \cdot E[T_{II}] + \Omega(1/N^2) \cdot E[T_{III}] + \Omega(1/N^2) \cdot (N + E[T_I]).$$

Simplifying this expression gives

$$\begin{aligned} E[T_{II}] &\geqslant \Omega(N) + \Omega(1/N) \cdot E[T_{III}] + \Omega(1/N) \cdot E[T_I] \\ &\geqslant \Omega(N) + \Omega(1/N) \cdot E[T_{III}] + \Omega(1/N) \cdot \big(\Omega(N^3) + E[T_{II}]\big) \\ &\geqslant \Omega(N^3) + \Omega(E[T_{III}]). \end{aligned}$$

Consider an altered process such that after reaching configuration (III), no vertex in P_L can be replaced (from death) until configuration (II) or (IV) is reached. This unilaterally decreases the fixation time of type 2.

Now, we bound the probability of reaching configuration (IV) from configuration (III) without first reaching configuration (II) in this altered process.

We examine the transition probabilities of a Markov Chain that tracks the number of type 2 individuals inside K_L. Then we examine the transition probabilities in a complete graph on n vertices where one type has higher fitness (i.e. the modified scenario). We show that the ratio between increasing and decreasing the number of type 2 individuals is higher in this modified scenario for every configuration. This will mean that the fixation probability in this modified scenario is an upper bound for the altered process.

Invincible process. When there are i type 2 individuals, we have two possibilities for the probabilities.

- If the bridgehead is occupied by type 2 individual, the probability that the number of type 2 individuals increases is

$$\frac{n-i}{n} \frac{i}{n-1}.$$

The probability that the number of type 2 individuals decreases is

$$\frac{1}{n} \frac{n-i}{n} + \frac{i-1}{n} \frac{n-i}{n-1}.$$

(Note that if the bridgehead dies, it has a smaller probability of being replaced by a type 1 individual.) The ratio between the probability of increase and the probability of decrease is

$$\frac{\frac{n-i}{n}\frac{i}{n-1}}{\frac{1}{n}\frac{n-i}{n}+\frac{i-1}{n}\frac{n-i}{n-1}} = \frac{i}{\frac{n-1}{n}+i-1} = \frac{i}{i-\frac{1}{n}} \leqslant 1+\frac{1}{n-1}\,.$$

– If the bridgehead is occupied by type 1 individual, the probability that the number of type 2 individuals increase is

$$\frac{1}{n}\frac{n-i-1}{n-1}+\frac{n-i-1}{n}\frac{i}{n}\,,$$

since when the bridgehead dies, it can be replaced by the individual on the path. The probability that the number of type 2 individuals decreases is

$$\frac{i}{n}\frac{n-i}{n-1}\,.$$

This gives us the ratio between the probability of increase and the probability of decrease

$$\frac{\frac{1}{n}\frac{n-i-1}{n-1}+\frac{n-i-1}{n}\frac{i}{n}}{\frac{i}{n}\frac{n-i}{n-1}} = \frac{\frac{n-1}{n}(i+1)+(n-i-1)i}{i(n-i)} = \frac{(1-\frac{1}{n})(1+\frac{1}{i})+n-i-1}{n-i} = 1+\frac{\frac{1}{i}-\frac{1}{n}-\frac{1}{ni}}{n-i} \leqslant 1+\frac{1}{ni} \leqslant 1+\frac{1}{n}\,.$$

Altered process. Now, let us imagine the altered scenario where the type 2 (later called mutants) has fitness $1+\frac{2}{n-1}$. The ratio between increasing and decreasing the number of mutants in configuration i is

$$\frac{\frac{n-i}{n}\frac{i(1+\frac{2}{n-1})}{F-1}}{\frac{i}{n}\frac{n-i}{F-1-\frac{2}{n}}}\,,$$

where F is the sum of the fitnesses of all mutants in the graph. This gives the ratio between the probability of increase and the probability of decrease

$$\frac{(1+\frac{2}{n-1})(F-1-\frac{2}{n})}{F-1} = 1+\frac{2}{n-1}-\frac{(1+\frac{2}{n-1})\frac{2}{n-1}}{F-1} \leqslant 1+\frac{2}{n-1}-(1+\frac{2}{n-1})\frac{2}{(n-1)^2} \leqslant 1+\frac{1}{n-1}\,,$$

where the last inequality holds for $n \geqslant 3$.

Now, the ratio between increasing and decreasing the number of mutants is higher than in every configuration of the original case. That means the fixation probability in this altered scenario is an upper bound for the invincible process. From §6.2 of [9], the fixation probability in this case is $\mathcal{O}(\frac{1}{n})$. This means that

$$E[T_{III}] \geqslant \Omega(1) + (1-\mathcal{O}(1/N)) \cdot E[T_{II}].$$

Simplifying yields

$$\begin{aligned} E[T_{III}] &\geqslant \Omega(1) + (1-\mathcal{O}(1/N)) \cdot \big(\Omega(N^3)+\Omega(E[T_{III}])\big) \\ &\geqslant \Omega(N^4). \end{aligned}$$

Putting all of the pieces together, we get

$$E[T_I] \geqslant \Omega(N^4).$$

This is what we wanted to prove. □

9 Bounds for any graph

In what follows, we use notation introduced in §1.1 of [1]. We let $\mathrm{AT}_{\tau,f,X_t}(G)$ be the expected absorption time of G (under a specified dynamic) starting at state X_t. We let $\mathrm{AT}_{r=1}(G)$ be the maximum expected bd absorption time over all possible initial mutant configurations when the fitness function is constant (i.e. $|f(V)|=1$), and there are only two types initially in the population (i.e. both $|\tau|=2$ and $X_0(V)=\tau$). Recall that $|X_t(V)|$ denotes the number of types at time t. (see Section 1).

9.1 Upper bound on expected absorption time under bd updating

Lemma 6. *Suppose $k \geqslant 2$ is an integer. Then $\lceil k/2 \rceil^2 \leqslant k^2/2$.*

Proof. If k is even then $\lceil k/2 \rceil^2 = k^2/4 \leqslant k^2/2$. If k is odd, it must be the case that $k \geqslant 3 > (\sqrt{2}-1)^{-1}$. Thus $1 + 1/k \leqslant \sqrt{2}$. Taking the logarithm of both sides gives us that $\log_2(k+1) - \log_2 k \leqslant 1/2$. By rearranging terms and multiplying both sides by 2, we get $2\log_2(k+1) - 1 \leqslant 2\log_2 k$ which means

$$(k+1)^2/4 \leqslant k^2/2. \tag{23}$$

Finally, we notice that when k is odd, $\lceil k/2 \rceil^2 = (k+1)^2/4$. □

In the following theorem, we show that the expected absorption time in the process with $|X_t(V)|$ types is at most $\log_2 |X_t(V)|$ longer than the process with only two types.

Theorem 7. *Let $G = (V, E)$ be a graph on N vertices. Let $|f(V)| = 1$. Then for bd updating, $\mathrm{AT}_{\tau,f,X_t}(G) \leqslant 2 \cdot \mathrm{AT}_{r=1}(G) \cdot \log_2 |X_t(V)|$ for all $t \geqslant 0$.*

Proof. We will map the multi-type Moran process to a two type Moran process. Clearly if $|X_t(V)| = 1$ the process has absorbed and thus its expected absorption time is upper bounded by $2 \cdot \mathrm{AT}_{r=1}(G) \cdot \log_2 |X_t(V)|$. Suppose $\mathrm{AT}_{\tau,f,X_t}(G) \leqslant 2 \cdot \mathrm{AT}_{r=1}(G) \cdot \log_2 k$ for all $X_t \in \Omega$ such that $|X_t(V)| < k$. Now consider the case when $|X_t(V)| = k > 1$. Let $\theta_{X_t} : X_t(V) \to \{\mathtt{A}, \mathtt{B}\}$ such that there is a subset of types $S \subseteq X_t(V)$ of size $\lceil X_t(V)/2 \rceil$ with $\theta_{X_t}(S) = \{\mathtt{A}\}$ and $\theta_{X_t}(X_t(V) \setminus S) = \{\mathtt{B}\}$. This is always possible since $X_t(V)$ is finite and thus under an arbitrary ordering of the elements of the set, we map the first $\lceil X_t(V)/2 \rceil$ elements to $\mathtt{A}$ and the rest to $\mathtt{B}$. Then we run the multi-type Moran process on $(\tau, f, \theta_{X_t} \circ X_t)$ until absorption which takes T_k steps. Notice that since $|(\theta_{X_t} \circ X_t)(V)| \leqslant 2$, this is the Moran process on a graph with two types under neutral evolution. When this process absorbs, it must be the case that $|(\theta_{X_t} \circ X_{t+T})(V)| = 1$ which in turn means

$$|X_{t+T_k}(V)| \leqslant \max\{|\theta_{X_t}(S)|, |\theta_{X_t}(V \setminus S)|\} \leqslant \lceil |X_t(V)|/2 \rceil = \lceil k/2 \rceil. \tag{24}$$

Thus by linearity of expectation we get

$$\mathrm{AT}_{\tau,f,X_t}(G) \leqslant \mathbb{E}[T_k] + \mathbb{E}\left[\mathrm{AT}_{\tau,f,X_{t+T_k}}(G)\right]. \tag{25}$$

Since $|X_{t+T_k}(V)| \leqslant \lceil k/2 \rceil < k$ for $k > 1$, the inductive hypothesis gives us

$$\mathbb{E}\left[\mathrm{AT}_{\tau,f,X_{t+T_k}}(G)\right] \leqslant 2 \cdot \mathrm{AT}_{r=1}(G) \cdot \log_2 \lceil k/2 \rceil. \tag{26}$$

Finally, we get

$$\begin{aligned}
\mathrm{AT}_{\tau,f,X_t}(G) &\leqslant \mathrm{AT}_{r=1}(G) + 2 \cdot \mathrm{AT}_{r=1}(G) \cdot \log_2 \lceil k/2 \rceil & (27)\\
&= 2 \cdot \mathrm{AT}_{r=1}(G) \cdot (1 + \log_2 \lceil k/2 \rceil^2) & (28)\\
&= \mathrm{AT}_{r=1}(G) \cdot \log_2 2\lceil k/2 \rceil^2 & (29)\\
&\leqslant 2 \cdot \mathrm{AT}_{r=1}(G) \cdot \log_2 k & (30)
\end{aligned}$$

where the last step follows from Lemma 6. □

Corollary 3. *Let $G = (V, E)$ be a graph on N vertices. Let $|f(V)| = 1$. Then for bd updating, $\mathrm{AT}_{\tau,f,X_t}(G) \leqslant 2N^6 \log_2 |X_t(V)|$ for all $t \geqslant 0$.*

Proof. By Corallary 12(i) of [10], we have $\mathrm{AT}_{r=1}(G) \leqslant \phi_G(V)^2 N^4 \leqslant N^6$. The result follows from Theorem 7. □

9.2 Lower bound on expected absorption time under bd updating

Theorem 8. *Let $G = (V, E)$ be a graph on N vertices. Let $|f(V)| = 1$. Fix $t \geqslant 0$ and let $k := |X_t(V)| > 1$. Then*

$$\mathrm{AT}_{\tau,f,X_t}(G) \geqslant N \cdot (1 - 1/k) \cdot H_{N\cdot(1-1/k)} \geqslant N \cdot (1 - 1/k) \ln(N \cdot (1 - 1/k)) \tag{31}$$

where $H_m := \sum_{i=1}^{m} 1/i$ is the mth harmonic number.

Proof. In order for absorption to occur on a graph with k types, it must be the case that at least $k - 1$ distinct vertices are selected for death since absorption only occurs when one type remains. Thus a lower bound on the absorption time is the expected amount of time for $k - 1$ vertices to be selected for death. Notice that the probability that a vertex $u \in V$ is selected for death is independent of f and t; let p_u be this probability. We have $p_u = T(u)/N$ where

$$T(u) := \sum_{v \in \Gamma(u)} \frac{1}{\deg(v)} \tag{32}$$

is the *temperature* of vertex $u \in V$.

We will make use of the coupon collector problem (see here [11]). For a probability vector $\pi \in [0,1]^m$, we denote CC_π as the number of coupon draws until each distinct type of m coupons has been drawn at least once, where π_i is the probability that the ith type of coupon is drawn. By considering deaths at vertices as coupons, we have that

$$\mathrm{AT}_{\tau,f,X_t}(G) \geqslant \min_{i \in X_t(V)} \mathsf{CC}_{\rho^{(-i)}} \tag{33}$$

where $\rho^{(-i)}$ is the $|V \setminus X_t^{-1}(i)|$-dimensional probability vector such that

$$\rho_u^{(-i)} \propto p_u \tag{34}$$

for each $u \in V \setminus X_t^{-1}(i)$; this is because (1) coupon draws are independent, and (2) the number of draws until a subset S of types of coupons have each been drawn is at least the number of draws *restricted to coupons whose types are in* S until the subset S of types of coupons have each been drawn. From Corollary 4.2 of [11], we have

$$\mathsf{CC}_\pi = \sum_{q=0}^{m-1} (-1)^{m-1-q} \sum_{\substack{J \subseteq [N] \\ |J|=q}} \left(1 - \sum_{j \in J} \pi_j\right)^{-1} \tag{35}$$

and $\mathsf{CC}_\pi = mH_m$ when π is the uniform distribution. By [12], $\mathsf{CC}_{\rho^{(-i)}}$ is Schur-convex and thus is minimized when $\rho^{(-i)}$ is uniform. Thus we have

$$\min_{i \in X_t(V)} \mathsf{CC}_{\rho^{(-i)}} \geqslant \min_{i \in X_t(V)} |V \setminus X_t^{-1}(i)| \cdot H_{|V \setminus X_t^{-1}(i)|}. \tag{36}$$

Since $\sum_{i \in X_t(V)} |X_t^{-1}(i)| = N$ and $|X_t^{-1}(i)| \geqslant 1$ for each $i \in X_t(V)$, we must have $\max_{i \in X_t(V)} \geqslant N/k$. This gives us

$$|V \setminus X_t^{-1}(i)| \geqslant N - \max_{j \in X_t(V)} |X_t^{-1}(j)| \geqslant N \cdot (1 - 1/k) \tag{37}$$

for each $i \in X_t(V)$. Finally, this means that

$$\min_{i \in X_t(V)} |V \setminus X_t^{-1}(i)| \cdot H_{|V \setminus X_t^{-1}(i)|} \geqslant N \cdot (1 - 1/k) \cdot H_{N \cdot (1-1/k)}. \tag{38}$$

It is known that $H_m \geqslant \ln m$ for every $m \geqslant 1$. □

9.3 Upper bound on expected absorption time under db updating

For a graph $G = (V, E)$, let $M := |E|$ be the number of edges, let $\deg_{\min} := \min \deg(u) \mid u \in V$ be the minimum degree of the vertices, and for $S \subseteq V$ let $\deg(S) := \sum_{u \in S} \deg(u)$. Let Φ be the *conductance* of G, defined as

$$\Phi := \min_{S \subseteq V} \frac{\#\{(u,v) \in E \mid u \in S \text{ and } v \notin S\}}{\min\{\deg(S), \deg(V \setminus S)\}} \tag{39}$$

where $0/0 = \infty$.

Theorem 9. *The diversity time of G under db updating is upper bounded by*

$$\mathcal{O}\left(\frac{MN \log N}{\Phi \cdot \deg_{\min}}\right). \tag{40}$$

Proof. See Theorem 2, Example 11 of [13] for the solution when there are only two initial types. When there are N initial types, we use the recursive reduction from Theorem 7 which adds a multiplicative logarithm factor based on the number of initial types. □

Corollary 4. *The diversity time of G under db updating is upper bounded by $\mathcal{O}(M^2 N \log N)$, which is at most $\mathcal{O}(N^5 \log N)$.*

Proof. The result follows from Theorem 9 since the conductance of a connected graph is at least $\Omega(1/M)$ and there are at most $\mathcal{O}(N^2)$ edges in a graph. □

9.4 Lower bound on expected absorption time under db updating

Theorem 10. *The diversity time of G under db updating is lower bounded by $\Omega(N \log N)$.*

Proof. The proof is similar to that of Theorem 8 though is simpler since the probability a death occurs at a vertex is exactly $1/N$. Thus the lower bound is precisely the solution to the classic coupon collector problem [11]. □

10 Directed graphs

First, we prove a general lemma about the upper bound on the diversity time on directed graph and then, we describe a contracting star: a graph that achieves that upper bound.

Lemma 7. *For all strongly connected directed graphs, the diversity time is at most* $2^{\mathcal{O}(N \log N)}$.

Proof. In any configuration, we have a probability of at least $\frac{1}{N^2}$ that type A replaces type B. In any configuration, there are at most $N-1$ individuals of type B (other than A). That means, after $N-1$ steps, the probability that the process is finished is at least $\left(\frac{1}{N^2}\right)^{N-1}$. That means, we can bound the diversity time as

$$T_N \leqslant N - 1 + \left(1 - \left(\frac{1}{N^2}\right)^{N-1}\right) T_N.$$

Rearranging, we get

$$T_N \leqslant N^{2(N-1)} \cdot (N-1) \leqslant N^{2N}.$$

That means the maximal expected time from any configuration is $2^{\mathcal{O}(N \log N)}$. □

10.1 Contracting stars

A *contracting star* graph consists of k blades and one central vertex. Every blade consists of n vertices indexed from 1 to n. For each blade,

- vertex i is bi-directionally connected to vertex $i+1$ for each $i = 1, \dots, n-1$;
- there is an edge from vertex i to the central vertex for each $i = 1, \dots, n$;
- there is an edge from vertex j to vertex i for each $1 \leqslant i < j \leqslant n$;
- there an edge from the central vertex to vertex 1.

The contracting star with k blades and n blade length has $N = kn + 1$ total vertices.

Lemma 8. *The diversity time for k types on a contracting star with k blades is* $2^{\Omega(n \log n)}/k$.

Proof. We lower bound the expected time until a vertex with index n is replaced in a blade. Since we treat all blades independently, this is also a lower bound on the diversity time. We suppose the blade is occupied by type 1 while the type 2 invades. To help the type 2 individuals, we suppose that all individuals of type 2 are invincible with the exception of the individual occupying the vertex with the highest index.

Let us look at the type 2 vertex with the highest index i.

- Under bd updating, type 2 spreads to index i with probability $\frac{1}{N}\frac{1}{i+1}$. But it is killed with probability $\frac{1}{N}\sum_{j>i}^{n}\frac{1}{j+1}$.

  For $\frac{1}{4}n \leqslant i \leqslant \frac{1}{2}n$, we have the probability of type 2 to be killed

  $$\frac{1}{N}\frac{1}{n}\sum_{j>i}^{n}\frac{1}{j+1} > \frac{1}{N}\sum_{j>i}^{2i}\frac{1}{2i+2} \geqslant \frac{1}{3N}.$$

  So the ratio between reproduction and killing is at most $\frac{12}{n}$, which means the type 2 is $n/12$ times more likely to die than to reproduce. That gives the expected time at least $(n/12)^{1/4n}$ for one blade.

- Under db updating, type 2 spreads to index i with probability $\frac{1}{N}\frac{1}{n-(i-1)}$.

  But it is killed with probability $\frac{1}{N}\frac{n-(i-1)-1}{n-(i-1)}$

  For $\frac{1}{4}n \leqslant i \leqslant \frac{1}{2}n$, we have the probability of type 2 to be killed is at least

  $$\frac{1}{N}\frac{n-(i-1)-1}{n-(i-1)} \geqslant \frac{1}{N}\left(1 - \frac{1}{n-(n/4-1)}\right) \geqslant 1/N$$

  So the ratio between reproduction and killing is at most $2/n$, which means the type 2 is $n/2$ times more likely to die than to reproduce. That gives the expected time at least $(n/2)^{1/4n}$ for one blade.

Since the processes are independent in blades, we have the total diversity time $2^{\Omega(n \log n)}/k$.

□

Corollary 5. *The diversity time on a contracting star with constant number of blades is* $2^{\Theta(n \log n)}$.

References

[1] Leslie Ann Goldberg, Marc Roth, and Tassilo Schwarz. Parameterised approximation of the fixation probability of the dominant mutation in the multi-type moran process. *Theoretical Computer Science*, page 114785, 2024.

[2] Erez Lieberman, Christoph Hauert, and Martin A Nowak. Evolutionary dynamics on graphs. *Nature*, 433(7023):312–316, 2005.

[3] Richard P Stanley. Enumerative combinatorics volume 1 second edition. *Cambridge studies in advanced mathematics*, 2011.

[4] Mark Broom, Christophoros Hadjichrysanthou, and Jan Rychtář. Evolutionary games on graphs and the speed of the evolutionary process. *Proceedings of the Royal Society A: Mathematical, Physical and Engineering Sciences*, 466(2117):1327–1346, 2010.

[5] David A Levin and Yuval Peres. *Markov chains and mixing times*, volume 107. American Mathematical Soc., 2017.

[6] Timo Kötzing and Martin S Krejca. First-hitting times under drift. *Theoretical Computer Science*, 796:51–69, 2019.

[7] Mark Broom and Jan Rychtář. An analysis of the fixation probability of a mutant on special classes of non-directed graphs. *Proceedings of the Royal Society A: Mathematical, Physical and Engineering Sciences*, 464:2609–2627, 2008.

[8] Christophoros Hadjichrysanthou, Mark Broom, and Jan Rychtár. Evolutionary games on star graphs under various updating rules. *Dynamic Games and Applications*, 1(3):386–407, 2011.

[9] Martin A Nowak. *Evolutionary Dynamics: Exploring the Equations of Life.* Harvard University Press, 2006.

[10] Josep Díaz, Leslie Ann Goldberg, George B Mertzios, David Richerby, Maria Serna, and Paul G Spirakis. Approximating fixation probabilities in the generalized moran process. *Algorithmica*, 69:78–91, 2014.

[11] Philippe Flajolet, Danièle Gardy, and Loïc Thimonier. Birthday paradox, coupon collectors, caching algorithms and self-organizing search. *Discrete Applied Mathematics*, 39:207–229, 1992.

[12] M Lawrence Clevenson and William Watkins. Majorization and the birthday inequality. *Mathematics Magazine*, 64(3):183–188, 1991.

[13] Colin Cooper and Nicolas Rivera. The linear voting model. In *43rd International Colloquium on Automata, Languages, and Programming (ICALP 2016)*. Schloss Dagstuhl-Leibniz-Zentrum fuer Informatik, 2016.

11 Additional Figures

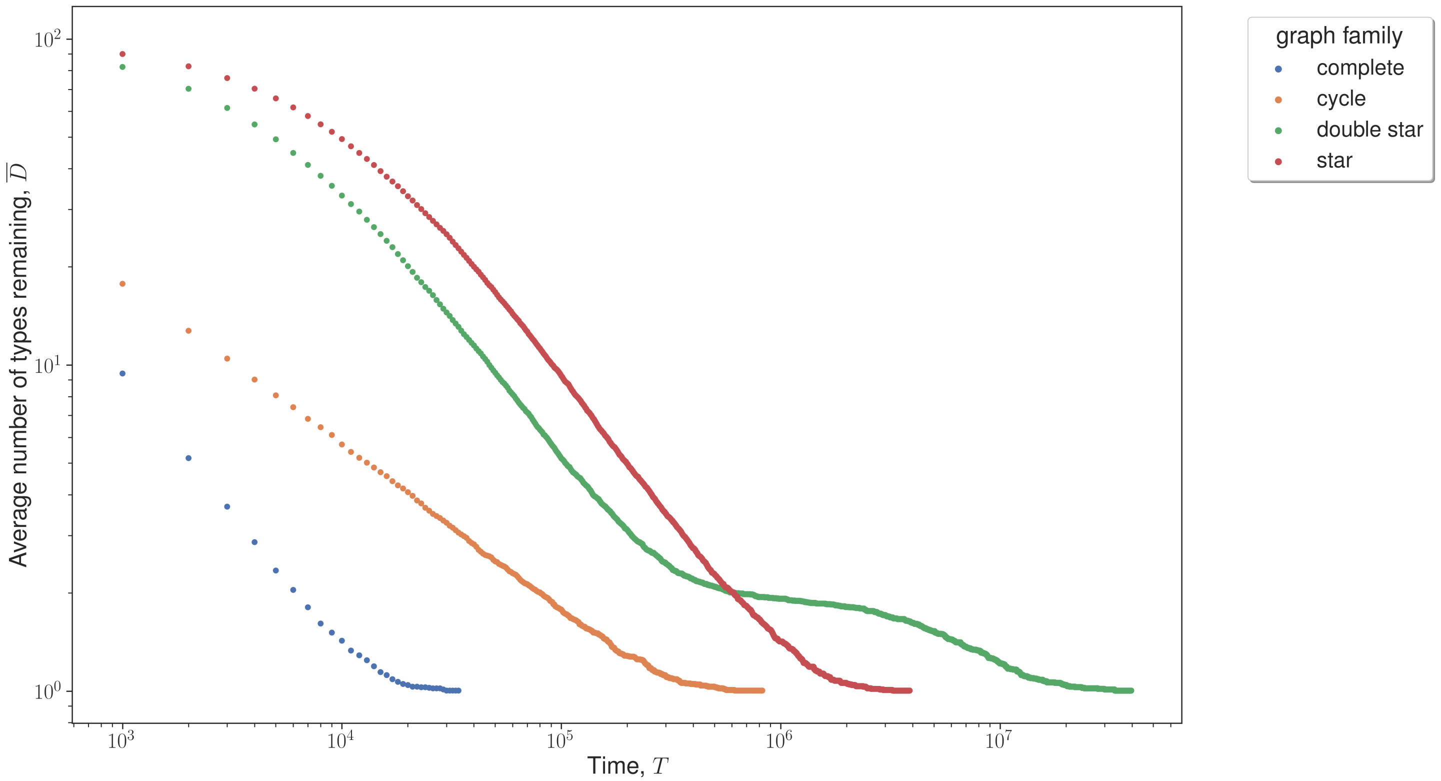

Figure S1: Results of average number of types remaining in the population, $\overline{D}$, at time T, averaged over 250 simulations of birth-death updating per graph. Each graph has $N = 100$ vertices. The plot is on a log-log scale. If $\overline{D} = 1$ at a particular time T, no dot is drawn. Only times that are multiples of 10^3 (excluding 0) are plotted.
